# Renal Function and Clinical Outcomes Among Elderly Patients With Nonvalvular Atrial Fibrillation From ANAFIE

**DOI:** 10.1016/j.jacasi.2023.02.002

**Published:** 2023-04-11

**Authors:** Wataru Shimizu, Takeshi Yamashita, Masaharu Akao, Hirotsugu Atarashi, Takanori Ikeda, Yukihiro Koretsune, Ken Okumura, Shinya Suzuki, Hiroyuki Tsutsui, Kazunori Toyoda, Atsushi Hirayama, Masahiro Yasaka, Takenori Yamaguchi, Satoshi Teramukai, Tetsuya Kimura, Yoshiyuki Morishima, Atsushi Takita, Hiroshi Inoue

**Affiliations:** a Department of Cardiovascular Medicine, Nippon Medical School, Tokyo, Japan; b Cardiovascular Institute, Tokyo, Japan; c Department of Cardiology, National Hospital Organization Kyoto Medical Center, Kyoto, Japan; d AOI Hachioji Hospital, Tokyo, Japan; e Department of Cardiovascular Medicine, Toho University Faculty of Medicine, Tokyo, Japan; f National Hospital Organization Osaka National Hospital, Osaka, Japan; g Division of Cardiology, Saiseikai Kumamoto Hospital Cardiovascular Center, Kumamoto, Japan; h Department of Cardiovascular Medicine, Faculty of Medical Sciences, Kyushu University, Fukuoka, Japan; i Department of Cerebrovascular Medicine, National Cerebral and Cardiovascular Center, Osaka, Japan; j Osaka Police Hospital, Osaka, Japan; k Department of Cerebrovascular Medicine and Neurology, Cerebrovascular Center, National Hospital Organization Kyushu Medical Center, Fukuoka, Japan; l Department of Biostatistics, Graduate School of Medical Science, Kyoto Prefectural University of Medicine, Kyoto, Japan; m Primary Medical Science Department, Daiichi Sankyo Co, Ltd, Tokyo, Japan; n Data Intelligence Department, Daiichi Sankyo Co, Ltd, Tokyo, Japan; o Saiseikai Toyama Hospital, Toyama, Japan

**Keywords:** anticoagulants, atrial fibrillation, elderly, renal function

## Abstract

**Background:**

Advancing age, decreasing renal function, and atrial fibrillation are strongly associated. Real-world evidence of direct oral anticoagulant (DOAC) use among elderly patients ≥75 years of age with nonvalvular atrial fibrillation and renal dysfunction is limited.

**Objectives:**

This study sought to assess 2-year outcomes and anticoagulant treatment, stratified by renal function.

**Methods:**

Enrolled patients were divided into 4 subgroups by creatinine clearance (CrCl) to determine the impact of renal dysfunction on clinical outcomes.

**Results:**

Of 32,275 patients, 26,202 with CrCl data were analyzed (median follow-up 2.00 [IQR: 1.92-2.00] years); 1.3% of patients had CrCl <15 mL/min, 10.7% had CrCl 15 to <30 mL/min, 33.4% had CrCl 30 to <50 mL/min, 35.8% had CrCl ≥50 mL/min, and 18.9% had unknown CrCl. Cumulative incidences of stroke/systemic embolic events, major bleeding, major plus clinically relevant nonmajor bleeding, cardiovascular death, all-cause death, and net clinical outcomes increased with decreasing CrCl. In multivariable Cox regression analysis, lower CrCl emerged as an independent risk factor for these clinical outcomes, except for major bleeding, compared with CrCl ≥50 mL/min. The effectiveness and safety of DOACs over warfarin were similar or better across 3 CrCl subgroups with CrCl 15 mL/min or more. DOAC use was associated with a lower risk of stroke/systemic embolic events, major bleeding, cardiovascular death, all-cause death, and net clinical outcome compared with warfarin in patients with CrCl 30 to <50 mL/min.

**Conclusions:**

Incidences of major clinical outcomes increased with decreasing renal function in elderly nonvalvular atrial fibrillation patients. DOACs were effective and safe even in patients with renal dysfunction (CrCl 15-<50 mL/min). (Prospective Observational Study in Late-Stage Elderly Patients with Non-Valvular Atrial Fibrillation: All Nippon AF In Elderly Registry [ANAFIE Registry]; UMIN000024006)

Advancing age, decreasing renal function, and atrial fibrillation (AF) are strongly associated.^1^ About one-third of patients with AF have chronic kidney disease (CKD). A close bidirectional relationship between these 2 conditions has been described, whereby CKD increases the risk of AF and AF increases the risk of CKD.^1^, ^2^, ^3^, ^4^

The age-related decline in renal function raises several issues for the medical care of patients with AF. Renal dysfunction is associated with poor outcomes, including increased risk of stroke/systemic embolic events (SEEs), major bleeding, and increased mortality.^5^, ^6^, ^7^, ^8^ Additionally, reduced renal function has a considerable effect on the pharmacokinetics, pharmacodynamics, and safety of drugs that have high renal excretion rates.^9^ Current clinical practice guidelines recommend the use of direct oral anticoagulants (DOACs), rather than warfarin, as the first-line therapy for stroke prevention in patients with nonvalvular atrial fibrillation (NVAF) and creatinine clearance (CrCl) ≥30 mL/min.^10^, ^11^, ^12^ However, DOACs such as dabigatran, rivaroxaban, apixaban, and edoxaban are excreted by the kidney to varying extents, ranging from 27% to 80%, and thus are more dependent on renal excretion compared with warfarin, which is primarily metabolized by the liver.^13^

There is a misconception that administering DOACs to elderly patients with renal dysfunction is challenging, and the practice of prescribing warfarin for these patients is deeply rooted in clinical settings. However, delayed warfarin metabolism is associated with an increased risk of bleeding, and increasing age is correlated with increased bleeding complications among patients taking warfarin.^14^^,^^15^ Compared with warfarin, DOACs appear to be equally safe and effective across all levels of CKD.^8^ At the same time, some studies have reported that DOACs were associated with a lower risk of all-cause mortality, major bleeding,^16^ and stroke/SEEs.^17^ In clinical trials, the relative efficacy and safety of DOACs compared with warfarin were consistent, regardless of the renal function level of patients.^18^, ^19^, ^20^ However, these clinical trials excluded patients with CrCl <25 mL/min or <30 mL/min, and data from patients with CrCl <30 mL/min are particularly limited.

Evidence of DOAC use among elderly patients with NVAF and severe renal dysfunction (CrCl <30 mL/min) is limited in real-world clinical practice, and it is important to accumulate such data. As such, the present subanalysis of the ANAFIE (All Nippon AF In the Elderly) registry, the largest NVAF registry worldwide, with more than 30,000 NVAF patients ≥75 years of age, assessed 2-year outcomes and anticoagulant treatment in elderly NVAF patients, stratified by renal function.

## Methods

### Study design

Details of the rationale and study design have been published previously.^21^ Briefly, the ANAFIE registry was a multicenter, prospective, observational study conducted between October 2016 and January 2020 and included 1,273 participating medical sites across Japan. The study complied with the Declaration of Helsinki; the locally appointed ethics committee approved the research protocol, and informed consent was obtained from the subjects (or their legally authorized representatives). The study was registered at UMIN Clinical Trials Registry under the unique identifier, UMIN000024006.

### Patients

Patients enrolled in the ANAFIE registry consisted of men and women ≥75 years of age at the time of providing informed consent who had been diagnosed with NVAF and could comply with study procedures and follow-up visits. Patients participating or planning to participate in other clinical studies, as well as those who had a history of artificial valve replacement, stroke, myocardial infarction, cardiac intervention, heart failure requiring hospitalization, or any bleeding leading to hospitalization within 1 month before enrollment; those with mitral stenosis; and those with a life expectancy of 1 year or less, were excluded from the registry.

For this subanalysis, patients enrolled in the ANAFIE registry were divided into 4 subgroups according to CrCl levels (<15, 15-<30, 30-<50, and ≥50 mL/min),^22^ estimated using the Cockcroft-Gault equation modified for Japanese individuals.^23^ As DOACs are not indicated for use in patients with CrCl <15 mL/min (kidney failure) in Japan, the 3 subgroups comprising patients with CrCl 15 to <30 mL/min (severe renal dysfunction), 30 to <50 mL/min (moderate renal dysfunction), and ≥50 mL/min (mild renal dysfunction or normal) were used for analysis of efficacy and safety of oral anticoagulants (OACs).

### Study outcomes and definitions

The incidence of stroke/SEEs was the primary endpoint. Secondary endpoints were the incidence of major bleeding, major plus clinically relevant nonmajor bleeding, minor bleeding, all bleeding, gastrointestinal bleeding, stroke, SEEs, ischemic stroke, hemorrhagic stroke, intracranial hemorrhage (ICH), and cardiovascular events (stroke, myocardial infarction, cardiac intervention for ischemic disease other than myocardial infarction, heart failure requiring hospitalization), cardiac events (myocardial infarction, cardiac intervention for ischemic disease other than myocardial infarction, heart failure requiring hospitalization, and cardiovascular death), ischemic heart disease, myocardial infarction, heart failure requiring hospitalization, cardiovascular death, cardiac death and sudden death, all-cause death, fractures, and falls. Net clinical outcomes consisted of stroke/SEEs, major bleeding, and all-cause death. Major bleeding was defined according to the classification of the International Society on Thrombosis and Haemostasis; detailed definitions are provided in the Supplemental Appendix. All events were adjudicated by independent blinded event evaluation committees.

Regarding the doses of DOACs, a reduced dose was a reduced on-label dose administered to a patient meeting the dose reduction criteria according to the prescribing information of each DOAC. An over-dose was a standard dose of DOAC that was administered despite the patient meeting the dose reduction criteria. An under-dose was a reduced on-label dose that was administered despite the patient meeting the standard-dose regimen criteria. An off-label dose was a dose lower than the reduced dose per the drug label.

### Statistical analysis

Continuous variables are reported using *P* values for trends calculated by the general linear model and categorical variables using *P* values calculated by the Cochran-Mantel-Haenszel correlation statistic. The 2-year cumulative incidences of clinical outcomes were estimated by Kaplan-Meier analysis among the 4 CrCl subgroups, and the log-rank test was used to compare the cumulative incidences of clinical outcomes between the subgroups. HRs for clinical outcomes were analyzed among the 4 CrCl subgroups with the CrCl ≥50 mL/min subgroup as the reference using the Cox proportional hazards model and using the Fine-Gray model with all-cause death as a competing risk. HRs were also analyzed for each clinical outcome in the CrCl 15 to <30 mL/min, 30 to <50 mL/min, or ≥50 mL/min subgroups according to the type of anticoagulant used (DOAC or warfarin), using the Cox proportional hazards model. A spline interpolation, with a model including creatinine clearance, anticoagulants (warfarin and DOAC), and creatinine clearance and anticoagulant interactions, was conducted to compare event rates between the warfarin and DOAC groups. As for the CrCl value, the natural cubic spline was used, the number of knots was set to 5, and the quantile levels were set to 0.05, 0.275, 0.5, 0.725, and 0.95. The same analysis as that described previously was performed for the analysis, excluding the off-label doses of DOACs. Statistical significance was confirmed by a 2-sided *P* value <0.05. SAS version 9.4 (SAS Institute) was used to conduct the analyses.

## Results

### Patient disposition and characteristics

**Figure 1:**
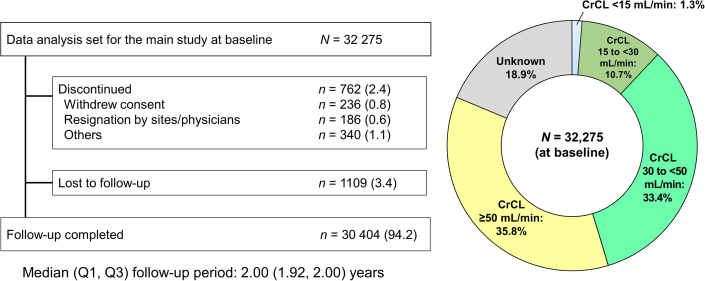
Patient Disposition and Distribution of CrCl Subgroups The figure is a flowchart depicting the patient disposition and a pie chart showing the distribution of creatinine clearance (CrCl) subgroups. Of the 32,275 patients included in the analysis set, 762 discontinued and 1,109 were lost to follow-up. In total, 30,404 patients completed a median follow-up of 2.00 (IQR: 1.92-2.00) years. By renal function, 404 (1.3%) of patients had CrCl <15 mL/min, 3,465 (10.7%) had CrCl 15 to <30 mL/min, 10,764 (33.4%) had CrCl 30 to <50 mL/min, 11,569 (35.8%) had CrCl ≥50 mL/min, and 6,102 (18.9%) had unknown CrCl.

Of the 32,275 patients included in the analysis set, 762 discontinued, and 1,109 were lost to follow-up. A total of 30,404 patients completed a median follow-up of 2.00 (IQR: 1.92-2.00) years. By renal function, 404 (1.3%) of patients had CrCl <15 mL/min, 3,465 (10.7%) had CrCl 15 to <30 mL/min, 10,764 (33.4%) had CrCl 30 to <50 mL/min, 11,569 (35.8%) had CrCl ≥50 mL/min, and 6,102 (18.9%) had unknown CrCl (Figure 1). After excluding patients with unknown CrCl, the total number of patients analyzed in this study was 26,202.

Regarding the main background characteristics of patients at the time of informed consent, patients had a mean age of 81.5 ± 4.8 years overall. Significant differences were observed in the distribution by renal function (trend *P* < 0.001). In the lower CrCl subgroups (<15 and 15-<30 mL/min), the proportion of female patients was higher than in the other subgroups, and patients in these lower CrCl subgroups were older, had lower body mass index, lower systolic and diastolic blood pressure, had higher CHADS_2_ /HAS-BLED scores, and had higher proportions of major bleeding history, comorbidities, and concomitant medications (including antiplatelet and antihypertensive drugs) compared with patients with CrCl 30 to <50 mL/min and ≥50 mL/min (Table 1).

**Table 1:** Background Characteristics of Patients by Renal Function (CrCl Level) at the Time of Informed Consent

	CrCl <15 mL/min or Dialysis (n = 404)	CrCl 15-<30 mL/min (n = 3,465)	CrCl 30-<50 mL/min (n = 10,764)	CrCl ≥50 mL/min (n = 11,569)	Overall (N = 32,275)	Trend *P* Value
Male	165 (40.8)	1,368 (39.5)	5,704 (53.0)	7,783 (67.3)	18,482 (57.3)	<0.001
Age, y	85.1 ± 6.0	85.6 ± 5.1	82.5 ± 4.6	79.3 ± 3.6	81.5 ± 4.8	<0.001
≥75-<80 y	87 (21.5)	453 (13.1)	3,083 (28.6)	6,758 (58.4)	12,895 (40.0)	<0.001
≥80-<85 y	106 (26.2)	975 (28.1)	4,101 (38.1)	3,725 (32.2)	10,961 (34.0)	
≥85-<90 y	109 (27.0)	1,251 (36.1)	2,813 (26.1)	979 (8.5)	6,295 (19.5)	
≥90-<95 y	79 (19.6)	655 (18.9)	699 (6.5)	103 (0.9)	1,848 (5.7)	
≥95-<100 y	20 (5.0)	129 (3.7)	67 (0.6)	4 (0.0)	265 (0.8)	
≥100 y	3 (0.7)	2 (0.1)	1 (0.0)	0 (0.0)	11 (0.0)	
BMI, kg/m^2^	21.1 ± 3.3	21.4 ± 3.3	22.6 ± 3.3	24.6 ± 3.5	23.3 ± 3.6	<0.001
SBP, mm Hg	124.0 ± 20.3	123.3 ± 19.2	126.5 ± 17.2	129.0 ± 16.0	127.4 ± 17.0	<0.001
DBP, mm Hg	66.7 ± 12.6	66.9 ± 12.2	69.9 ± 11.8	72.2 ± 11.1	70.6 ± 11.6	<0.001
CrCl, mL/min	11.7 ± 5.8	24.2 ± 4.1	40.6 ± 5.7	64.0 ± 14.1	48.4 ± 18.2	-
eGFR, mL/min/1.73 m^2^	15.3 ± 8.0	32.6 ± 8.4	48.9 ± 10.2	66.0 ± 15.6	53.8 ± 17.7	<0.001
CHADS_2_ score	3.3 ± 1.2	3.2 ± 1.2	2.9 ± 1.2	2.7 ± 1.2	2.9 ± 1.2	<0.001
HAS-BLED score	2.7 ± 0.9	2.2 ± 0.9	1.9 ± 0.9	1.7 ± 0.8	1.9 ± 0.9	<0.001
History of major bleeding	37 (9.2)	203 (5.9)	499 (4.6)	470 (4.1)	1,439 (4.5)	<0.001
AF type						
Paroxysmal	151 (37.4)	1,216 (35.1)	4,543 (42.2)	4,961 (42.9)	13,586 (42.1)	<0.001
Persistent	62 (15.3)	598 (17.3)	1,697 (15.8)	1,897 (16.4)	5,336 (16.5)	
Long-standing persistent/permanent	191 (47.3)	1,651 (47.6)	4,524 (42.0)	4,711 (40.7)	13,353 (41.3)	
Catheter ablation	20 (5.0)	156 (4.5)	848 (7.9)	1,295 (11.2)	2,970 (9.2)	<0.001
Use of anticoagulants						
Warfarin	237 (58.7)	1,291 (37.3)	2,664 (24.7)	2,446 (21.1)	8,233 (25.5)	<0.001
TTR	63.9 ± 33.9	69.8 ± 31.0	75.9 ± 29.5	78.1 ± 28.5	75.5 ± 29.8	<0.001
DOACs	88 (21.8)	1,869 (53.9)	7,360 (68.4)	8,380 (72.4)	21,585 (66.9)	<0.001
Standard dose	0 (0.0)	9 (0.3)	503 (4.7)	3,116 (26.9)	3,826 (11.9)	<0.001
Over-dose	0 (0.0)	36 (1.0)	425 (3.9)	206 (1.8)	698 (2.2)	
Reduced dose	5 (1.2)	1,550 (44.7)	5,295 (49.2)	2,192 (18.9)	9,548 (29.6)	
Under dose	3 (0.7)	65 (1.9)	826 (7.7)	2,633 (22.8)	3,630 (11.2)	
Off-label dose	0 (0.0)	154 (4.4)	309 (2.9)	227 (2.0)	795 (2.5)	
Nonoral anticoagulants	4 (1.0)	0 (0.0)	3 (0.0)	4 (0.0)	12 (0.0)	0.003
No use	75 (18.6)	305 (8.8)	737 (6.8)	739 (6.4)	2,445 (7.6)	<0.001
Concomitant medications	386 (95.5)	3,365 (97.1)	10,332 (96.0)	10,954 (94.7)	30,637 (94.9)	<0.001
Antiplatelet drugs	103 (26.7)	717 (21.3)	1,995 (19.3)	1,927 (17.6)	5,704 (18.6)	<0.001
Comorbidities						
Hypertension	311 (77.0)	2,698 (77.9)	8,185 (76.0)	8,661 (74.9)	24,312 (75.3)	<0.001
Diabetes mellitus	116 (28.7)	903 (26.1)	2,966 (27.6)	3,401 (29.4)	8,733 (27.1)	<0.001
Chronic kidney disease	156 (38.6)	1,745 (50.4)	3,070 (28.5)	969 (8.4)	6,705 (20.8)	<0.001
Myocardial infarction	34 (8.4)	296 (8.5)	674 (6.3)	560 (4.8)	1,851 (5.7)	<0.001
Heart failure	248 (61.4)	2,181 (62.9)	4510 (41.9)	3,328 (28.8)	12,116 (37.5)	<0.001
History of cerebrovascular disease	119 (29.5)	879 (25.4)	2,571 (23.9)	2,378 (20.6)	7,303 (22.6)	<0.001
Gastrointestinal disease	103 (25.5)	1,144 (33.0)	,3289 (30.6)	3,221 (27.8)	9,467 (29.3)	<0.001
Active cancer	44 (10.9)	391 (11.3)	1,246 (11.6)	1,255 (10.8)	3,569 (11.1)	0.276
Dementia	57 (14.1)	493 (14.2)	992 (9.2)	530 (4.6)	2,512 (7.8)	<0.001
Fall within 1 y	58 (14.4)	431 (12.4)	838 (7.8)	632 (5.5)	2,347 (7.3)	<0.001

Values are n (%) or mean ± SD.

AF = atrial fibrillation; BMI = body mass index; CHADS_2_ = congestive heart failure/LV dysfunction, hypertension, age ≥75 years, diabetes mellitus, prior stroke or transient ischemic attack; CrCl = creatinine clearance; DBP = diastolic blood pressure; DOAC = direct oral anticoagulant; eGFR = estimated glomerular filtration rate; HAS-BLED = hypertension, abnormal renal or liver function, stroke, bleeding, labile international normalized ratio, elderly, drugs or alcohol; SBP = systolic blood pressure; TTR = time in therapeutic range.

A higher proportion of patients with CrCl <15 mL/min were prescribed warfarin (58.7%) vs DOACs (21.8%) compared with patients with other CrCl subgroups.

### Relationship between CrCl and event rates

The Central Illustration shows the cumulative incidence rate of the main outcomes in the 4 subgroups. The cumulative incidences of all outcomes (ie, stroke/SEEs, major bleeding, major plus clinically relevant nonmajor bleeding, cardiovascular death, all-cause death, and net clinical outcomes) increased as the CrCl decreased. The most pronounced numerical increases in the cumulative incidences at 2 years were observed for all-cause death (3.8% and 17.2% in the CrCl ≥50 mL/min and 15-<30 mL/min groups, respectively) and net clinical outcomes (6.9% and 20.7%, respectively). Of note, the incidence of major bleeding events and ICH tended to be less affected by differences in CrCl.

**Central Illustration:**
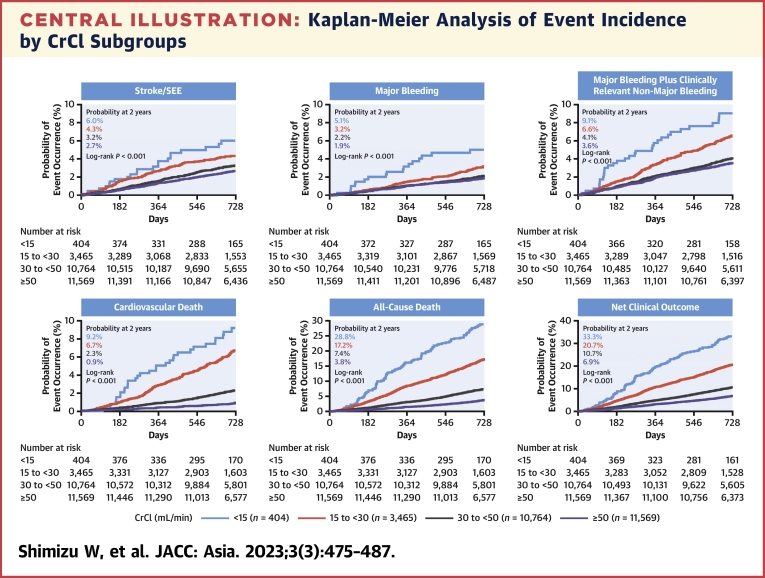
Kaplan-Meier Analysis of Event Incidence by CrCl Subgroups Kaplan-Meier curves show that cumulative incidences of all outcomes, stroke/systemic embolic events (SEEs), major bleeding, major plus clinically relevant nonmajor bleeding, cardiovascular death, all-cause death, and net clinical outcomes increased as creatinine clearance (CrCl) decreased. All-cause death and net clinical outcomes were the most marked numerical increases in the cumulative incidences at 2 years.

Table 2 and Supplemental Table 1 show the univariable and multivariable analysis of main outcomes among the 4 renal function subgroups. In the univariable analysis (Table 2), the risk of all events, including all-cause death and net clinical outcome, was significantly greater in the CrCl <15 mL/min and CrCl 15 to <30 mL/min subgroups compared with CrCl ≥50 mL/min, except for ICH in the CrCl <15 mL/min group. Similar findings were observed in the outcomes of the CrCl 30 to <50 mL/min group, except for major bleeding, ICH, major plus clinically relevant nonmajor bleeding, and gastrointestinal bleeding.

**Table 2:** Univariable and Multivariable Analyses of Main Outcomes by Renal Function

Event by CrCl	n	Events	Univariable		Multivariable		
			HR (95% CI)	*P* Value	HR (95% CI)	*P* Value	Trend *P* Value
Stroke/SEEs							0.053
<15 mL/min	404	21 (5.2)	2.35 (1.51-3.66)	<0.001	1.59 (0.99-2.56)	0.053	—
15-<30 mL/min	3,465	137 (4.0)	1.67 (1.36-2.04)	<0.001	1.31 (1.02-1.67)	0.032	—
30-<50 mL/min	10,764	329 (3.1)	1.21 (1.04-1.42)	0.017	1.10 (0.92-1.30)	0.298	—
≥50 mL/min	11,569	299 (2.6)	ref	—	ref	—	—
Major bleeding							0.232
<15 mL/min	404	18 (4.5)	2.93 (1.81-4.75)	<0.001	1.61 (0.96-2.72)	0.074	—
15-<30 mL/min	3,465	96 (2.8)	1.67 (1.32-2.13)	<0.001	1.12 (0.84-1.50)	0.439	—
30-<50 mL/min	10,764	216 (2.0)	1.14 (0.94-1.38)	0.174	0.95 (0.77-1.16)	0.605	—
≥50 mL/min	11,569	208 (1.8)	ref	—	ref	—	—
Intracranial hemorrhage							0.745
<15 mL/min	404	8 (2.0)	1.76 (0.87-3.59)	0.117	0.98 (0.46-2.07)	0.951	—
15-<30 mL/min	3,465	57 (1.7)	1.36 (1.00-1.84)	0.049	0.95 (0.66-1.37)	0.784	—
30-<50 mL/min	10,764	153 (1.4)	1.11 (0.88-1.38)	0.379	0.94 (0.73-1.20)	0.593	—
≥50 mL/min	11,569	152 (1.3)	ref	—	ref	—	—
Major plus clinically relevant nonmajor bleeding							0.002
<15 mL/min	404	32 (7.9)	2.72 (1.90-3.90)	<0.001	1.80 (1.22-2.65)	0.003	
15-<30 mL/min	3,465	204 (5.9)	1.86 (1.57-2.20)	<0.001	1.34 (1.09-1.65)	0.005	
30-<50 mL/min	10,764	417 (3.9)	1.15 (1.00-1.31)	0.051	0.99 (0.85-1.15)	0.920	
≥50 mL/min	11,569	400 (3.5)	ref		ref		
All bleeding							0.037
<15 mL/min	404	54 (13.4)	2.21 (1.68-2.91)	<0.001	1.53 (1.14-2.07)	0.005	—
15-<30 mL/min	3,465	347 (10.0)	1.50 (1.33-1.70)	<0.001	1.13 (0.97-1.32)	0.107	—
30-<50 mL/min	10,764	848 (7.9)	1.11 (1.01-1.22)	0.036	0.98 (0.88-1.09)	0.672	—
≥50 mL/min	11,569	842 (7.3)	ref	—	ref	—	—
Gastrointestinal bleeding							0.090
<15 mL/min	404	26 (6.4)	2.32 (1.56-3.45)	<0.001	1.65 (1.07-2.55)	0.023	
15-<30 mL/min	3,465	160 (4.6)	1.54 (1.28-1.86)	<0.001	1.14 (0.91-1.43)	0.250	
30-<50 mL/min	10,764	391 (3.6)	1.15 (0.99-1.32)	0.060	1.01 (0.87-1.18)	0.859	
≥50 mL/min	11,569	375 (3.2)	ref		ref		
Cardiovascular death							<0.001
<15 mL/min	404	32 (7.9)	11.54 (7.73-17.22)	<0.001	5.68 (3.63-8.88)	<0.001	
15-<30 mL/min	3,465	206 (6.0)	7.96 (6.24-10.15)	<0.001	4.35 (3.25-5.83)	<0.001	
30-<50 mL/min	10,764	226 (2.1)	2.62 (2.06-3.33)	<0.001	2.03 (1.57-2.62)	<0.001	
≥50 mL/min	11,569	95 (0.8)					
All-cause death							<0.001
<15 mL/min	404	113 (28.0)	9.19 (7.46-11.31)	<0.001	4.46 (3.53-5.64)	<0.001	—
15-<30 mL/min	3,465	568 (16.4)	4.96 (4.37-5.63)	<0.001	2.72 (2.33-3.18)	<0.001	—
30-<50 mL/min	10,764	763 (7.1)	2.01(1.78-2.26)	<0.001	1.51 (1.33-1.72)	<0.001	—
≥50 mL/min	11,569	419 (3.6)	ref	—	ref	—	—
Net clinical outcome							<0.001
<15 mL/min	404	131 (32.4)	5.83 (4.85-7.02)	<0.001	3.11 (2.53-3.82)	<0.001	—
15-<30 mL/min	3,465	691 (19.9)	3.28 (2.96-3.63)	<0.001	2.00 (1.77-2.27)	<0.001	—
30-<50 mL/min	10,764	1,112 (10.3)	1.59 (1.45-1.74)	<0.001	1.26 (1.14-1.40)	<0.001	—
≥50 mL/min	11,569	773 (6.7)	ref	—	ref	—	—

Values are n (%), unless otherwise indicated. The CrCl ≥50 subgroup served as the reference.

Sex, age, body mass index, history of major bleeding, atrial fibrillation type, hypertension, severe hepatic dysfunction, diabetes mellitus, hyperuricemia, heart disease (heart failure, left ventricular systolic dysfunction), myocardial infarction, cerebrovascular disease, thrombosis/embolism-related disease, active cancer, dementia, fall within 1 year, anticoagulants, catheter ablation, antiarrhythmics, antiplatelet drugs, proton pump inhibitors, P-glycoprotein inhibitors, dyslipidemia, gastrointestinal disease, and polypharmacy were included in the model.

CrCl = creatinine clearance; SEE = systemic embolic event(s).

In the multivariable Cox proportional hazards model (Table 2, Supplemental Table 1), there was a significantly higher risk of stroke/SEE only in the CrCl 15 to <30 mL/min subgroup. No differences were observed in the risk of major bleeding or ICH in any of the 3 CrCl <50 mL/min subgroups compared with the CrCl ≥50 mL/min subgroup. However, a significantly higher risk of major plus clinically relevant nonmajor bleeding was observed in the CrCl <15 mL/min and CrCl 15 to <30 mL/min subgroups, and the risk of all bleeding and gastrointestinal bleeding was also higher in the CrCl <15 mL/min subgroup compared with the CrCl ≥50 mL/min subgroup. Significant differences were observed in the 3 CrCl <50 mL/min subgroups compared with the CrCl ≥50 mL/min subgroup for cardiovascular death, all-cause death, and net clinical outcome (Table 2). The risk of cardiac events, ischemic heart disease, myocardial infarction, heart failure requiring hospitalization, cardiac death and sudden death, and cardiovascular events was significantly higher in the 3 CrCl <50 mL/min subgroups than in the CrCl ≥50 mL/min subgroup. In general, the risk of fractures and falls was significantly higher in the subgroups with lower CrCl than with CrCl ≥50 mL/min (Supplemental Table 1).

We conducted additional analyses by renal function, applying the Fine-Gray model and using all-cause death as a competing risk. The results of this analysis revealed that after incorporating the competing risk into the statistical analysis, the association between renal function and events overall did not change (Supplemental Table 2).

### Relationship between anticoagulation status and event rates by CrCl levels

The distribution of DOAC dose and prothrombin time–international normalized ratio and time in therapeutic range of warfarin by renal function is shown in Supplemental Table 3. As noted previously, the CrCl <15 mL/min subgroup was not included in the analysis due to DOAC use contraindications. In the CrCl ≥50 mL/min subgroup, 37.2%, 26.2%, and 31.4% of patients received the standard dose, reduced dose, and under-dose, respectively. In the CrCl 30 to <50 mL/min and CrCl 15 to <30 mL/min subgroups, 71.9% and 82.9% of patients received the reduced dose, respectively. Prothrombin time–international normalized ratio and time in therapeutic range of warfarin were higher in the CrCl ≥50 mL/min subgroup.

Table 3 and Supplemental Table 4 summarize the main results of multivariable analyses of risks of outcomes in the CrCl 15 to <30 mL/min, CrCl 30 to <50 mL/min, and CrCl ≥50 mL/min subgroups treated with DOACs vs warfarin. In patients with CrCl ≥50 mL/min, the DOAC group had a significantly lower risk of stroke/SEEs, major plus clinically relevant nonmajor bleeding, fractures, and falls than the warfarin group. In patients with CrCl 30 to <50 mL/min, the DOAC group had significantly lower risks of stroke/SEEs, stroke, ischemic stroke, major bleeding, ICH, cardiovascular death, all-cause death, net clinical outcome, cardiac events, cardiovascular events, heart failure requiring hospitalization, and fractures than did the warfarin group. In patients with CrCl 15 to <30 mL/min, the DOAC group had a significantly lower risk of cardiac events, heart failure requiring hospitalization, and cardiovascular events than the warfarin group, but there was no difference in the risk of stroke/SEEs and major bleeding between the DOAC and warfarin groups.

**Table 3:** Multivariable Analyses of Main Outcomes by Renal Function and Type of Oral Anticoagulant

Event	n	CrCl 15-<30mL/min			n	CrCl 30-<50 mL/min			n	CrCl ≥50 mL/min		
		Events	HR (95% CI)	*P* Value		Events	HR (95% CI)	*P* Value		Events	HR (95% CI)	*P* Value
Stroke/SEEs												
Warfarin	1,291	51 (4.0)	ref	—	2,664	101 (3.8)	ref	—	2,446	83 (3.4)	ref	—
No OAC use	305	18 (5.9)	1.80 (1.01-3.20)	0.047	737	28 (3.8)	1.19 (0.77-1.86)	0.432	739	16 (2.2)	0.94 (0.53-1.64)	0.817
DOAC	1,869	68 (3.6)	0.89 (0.61-1.29)	0.541	7,360	200 (2.7)	0.75 (0.59-0.96)	0.024	8,380	200 (2.4)	0.76 (0.58-0.98)	0.037
Major bleeding												
Warfarin	1,291	45 (3.5)	ref	—	2,664	74 (2.8)	ref	—	2,446	51 (2.1)	ref	—
No OAC use	305	7 (2.3)	0.65 (0.28-1.48)	0.306	737	15 (2.0)	0.74 (0.42-1.33)	0.319	739	5 (0.7)	0.36 (0.14-0.91)	0.032
DOAC	1,869	44 (2.4)	0.67 (0.43-1.03)	0.065	7,360	127 (1.7)	0.64 (0.48-0.86)	0.003	8,380	152 (1.8)	0.91 (0.66-1.26)	0.572
Intracranial hemorrhage												
Warfarin	1,291	26 (2.0)	ref	—	2,664	56 (2.1)	ref	—	2,446	40 (1.6)	ref	—
No OAC use	305	5 (1.6)	0.77 (0.28-2.10)	0.612	737	7 (1.0)	0.50 (0.22-1.13)	0.096	739	5 (0.7)	0.43 (0.16-1.11)	0.080
DOAC	1,869	26 (1.4)	0.64 (0.36-1.12)	0.115	7,360	90 (1.2)	0.60 (0.43-0.85)	0.004	8,380	107 (1.3)	0.79 (0.54-1.14)	0.213
Major plus clinically relevant nonmajor bleeding												
Warfarin	1,291	86 (6.7)	ref	—	2,664	112 (4.2)	ref	—	2,446	107 (4.4)	ref	—
No OAC use	305	15 (4.9)	0.80 (0.46-1.38)	0.419	737	25 (3.4)	0.81 (0.52-1.25)	0.340	739	9 (1.2)	0.28 (0.14-0.55)	<0.001
DOAC	1,869	103 (5.5)	0.82 (0.62-1.10)	0.186	7,360	280 (3.8)	0.90 (0.72-1.12)	0.336	8,380	284 (3.4)	0.77 (0.62-0.96))	0.021
All bleeding												
Warfarin	1,291	135 (10.5)	ref	—	2,664	233 (8.8)	ref	—	2,446	212 (8.7)	ref	—
No OAC use	305	24 (7.9)	0.70 (0.44-1.09)	0.113	737	42 (5.7)	0.65 (0.47-0.92)	0.014	739	19 (2.6)	0.30 (0.19-0.49)	< 0.001
DOAC	1,869	188 (10.1)	0.96 (0.76-1.20)	0.719	7,360	573 (7.8)	0.92 (0.79-1.08)	0.295	8,380	611 (7.3)	0.86 (0.73-1.01)	0.068
Gastrointestinal bleeding												
Warfarin	1,291	61 (4.7)	ref	—	2,664	94 (3.5)	ref	—	2,446	91 (3.7)	ref	—
No OAC use	305	8 (2.6)	0.52 (0.24-1.11)	0.090	737	27 (3.7)	1.04 (0.67-1.63)	0.859	739	13 (1.8)	0.50 (0.28-0.91)	0.023
DOAC	1,869	91 (4.9)	1.10 (0.78-1.54)	0.584	7,360	270 (3.7)	1.10 (0.87-1.40)	0.422	8,380	271 (3.2)	0.91 (0.71-1.16)	0.455
Cardiovascular death												
Warfarin	1,291	74 (5.7)	ref	—	2,664	78 (2.9)	ref	—	2,446	27 (1.1)	ref	—
No OAC use	305	23 (7.5)	1.61 (0.98-2.63)	0.058	737	19 (2.6)	1.03 (0.61-1.74)	0.923	739	5 (0.7)	0.95 (0.35-2.60)	0.928
DOAC	1,869	109 (5.8)	1.11 (0.82-1.50)	0.510	7,360	129 (1.8)	0.67 (0.50-0.90)	0.007	8,380	63 (0.8)	0.84 (0.53-1.33)	0.452
All-cause death												
Warfarin	1,291	204 (15.8)	ref	—	2,664	242 (9.1)	ref	—	2,446	95 (3.9)	ref	—
No OAC use	305	72 (23.6)	1.73 (1.31-2.30)	<0.001	737	62 (8.4)	1.08 (0.81-1.45)	0.586	739	31 (4.2)	1.38 (0.90-2.11)	0.140
DOAC	1,869	292 (15.6)	1.05 (0.88-1.27)	0.586	7,360	459 (6.2)	0.74 (0.63-0.87)	<0.001	8,380	293 (3.5)	1.01 (0.80-1.28)	0.915
Net clinical outcome												
Warfarin	1,291	259 (20.1)	ref	—	2,664	347 (13.0)	ref	—	2,446	181 (7.4)	ref	—
No OAC use	305	85 (27.9)	1.60 (1.24-2.08)	<0.001	737	87 (11.8)	1.09 (0.85-1.40)	0.490	739	48 (6.5)	1.12 (0.81-1.57)	0.488
DOAC	1,869	347 (18.6)	0.96 (0.82-1.14)	0.665	7,360	678 (9.2)	0.75 (0.66-0.86)	<0.001	8,380	544 (6.5)	0.96 (0.81-1.14)	0.613

Warfarin group served as the reference.

Confounding variables included in the model are as listed in Table 2, excluding anticoagulants.

Abbreviations as in Tables 1 and 2.

No OAC use significantly increased the risk of stroke/SEEs, all-cause death, and net clinical outcome compared with warfarin in patients with CrCl 15 to <30 mL/min (Table 3). Among patients with CrCl 30 to <50 mL/min and ≥50 mL/min, the risk of minor bleeding was significantly lower in the no OAC group compared with the warfarin group; among patients with CrCl 30 to <50 mL/min, the risk of cardiac events was significantly lower in the no OAC group compared with the warfarin group; and among patients with CrCl ≥50 mL/min, the risk of fractures and falls was significantly lower in the no OAC group compared with the warfarin group (Supplemental Table 4).

Supplemental Table 5 indicates that the results of multivariable analyses by renal function and type of anticoagulant (excluding off-label doses of DOACs, n = 233) are similar to those shown in Supplemental Table 4. In the CrCl 30 to <50 mL/min subgroup, DOACs reduced the risk of stroke/SEEs, stroke, ischemic stroke, major bleeding, ICH, cardiac events, heart failure requiring hospitalization, cardiovascular events, cardiovascular death, all-cause deaths, fractures, and net clinical outcomes vs warfarin. In the CrCl ≥50 mL/min subgroup, DOACs reduced the risk of stroke/SEEs, major plus clinically relevant nonmajor bleeding, fractures, and falls vs warfarin.

**Figure 2:**
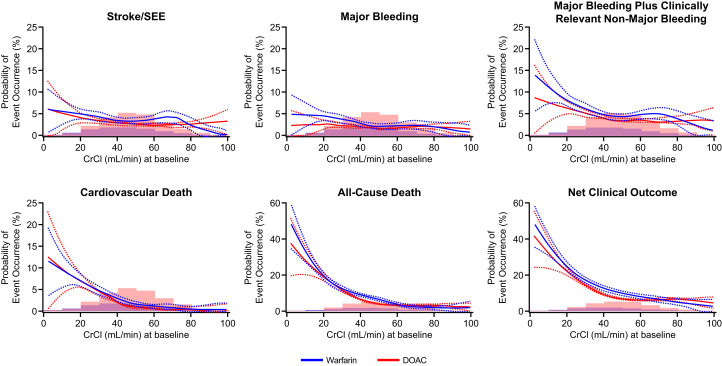
Renal Function, Event Incidence by Oral Anticoagulants, and Risk Assessment The Kaplan-Meier curves show that with declining renal function, the risk of major plus clinically relevant nonmajor bleeding, cardiovascular death, all-cause death, and net clinical outcomes remarkably increased in both direct oral anticoagulant (DOAC) and warfarin groups, but it had a lower impact on the risk of stroke/systemic embolic events (SEEs) and major bleeding. Histograms below the Kaplan-Meier curves show the spline curve risk assessment for stroke/SEEs, major bleeding, major plus clinically relevant major bleeding, death from cardiovascular disorders, all-cause deaths, and net clinical outcome. **Solid lines** denote the estimated probability of event occurrence; **dashed lines** denote the upper and lower limits of the 95% CI. CrCl = creatinine clearance.

Figure 2 shows the correlation between CrCl and the incidence of event occurrence by OAC. The results indicate that with declining renal function, the risk of major plus clinically relevant nonmajor bleeding, cardiovascular death, all-cause death, and net clinical outcomes remarkably increased in both DOAC and warfarin groups. However, the impact of renal function decline less obvious on the risk of stroke/SEEs and major bleeding.

## Discussion

### Major findings

First, we found that 45.3% of elderly patients with NVAF from the ANAFIE registry had moderate to severe renal dysfunction (CrCl <50 mL/min). Second, the cumulative incidences of stroke/SEEs, major bleeding, major plus clinically relevant nonmajor bleeding, cardiovascular death, all-cause death, and net clinical outcomes increased as the CrCl decreased, with more marked increases occurring for all-cause death and, consequently, net clinical outcomes. Multivariable Cox regression analysis showed that lower CrCl emerged as an independent risk factor for these clinical outcomes except for major bleeding compared with CrCl ≥50 mL/min. Third, DOACs showed at least similar effectiveness and safety compared with warfarin, irrespective of CrCl levels (15 mL/min or more), including patients with CrCl 15 to <30 mL/min who were excluded from the phase 3 clinical trials of DOACs. DOAC use was associated with a lower risk of stroke/SEEs, major bleeding, ICH, cardiovascular events, heart failure requiring hospitalization, cardiovascular death, all-cause death, net clinical outcome, fractures, and falls compared with warfarin in patients with CrCl 30 to <50 mL/min. No OAC use increased the risk of stroke/SEEs, all-cause death, and net clinical outcome vs warfarin in patients with CrCl 15 to <30 mL/min.

### Prevalence of renal dysfunction

The present proportion of elderly patients with NVAF and moderate to severe renal dysfunction (CrCl <50 mL/min) was similar to that in a registry of patients with NVAF >75 years of age who were hospitalized for any cause in internal medicine departments in Spain (43.8% meeting the criteria for CKD).^24^ A previous Japanese registry, the J-RHYTHM registry, showed that the percentage of elderly NVAF patients with CrCl <80 mL/min was higher at 60.8%,^7^ but the difference is likely due to the different cutoff value used for CrCl. Another registry, the Fushimi AF registry, showed that the percentage of NVAF patients with CrCl <50 mL/min was 37%.^5^

### Relationship between CrCl and event rates

Considering that DOACs are not indicated for patients with CrCl <15 mL/min, in the present study, we included the CrCl <15 mL/min subgroup only to investigate the effect of severe renal dysfunction on the clinical outcomes; the remaining subgroups (CrCl 15-<30, 30-<50, and ≥50 mL/min) were used for the analysis of effectiveness and safety of OACs. In line with previous reports,^8^ our data demonstrated that the cumulative incidences of all major outcomes increased as renal function decreased. In subgroups with CrCl <15, 15 to <30, and 30 to <50 mL/min, the risks of stroke/SEEs, major plus clinically relevant nonmajor bleeding, cardiovascular events, cardiovascular death, all-cause death, and net clinical outcome were significantly higher compared with those in the CrCl ≥50 mL/min subgroup. These findings from a large-scale registry of elderly patients with NVAF highlight the need to undergo appropriate management to prevent such events in these patients.

In contrast with previous findings,^5^^,^^8^^,^^25^ the multivariable analysis suggested that impaired renal function did not increase the risk of major bleeding in the ANAFIE registry population; however, the risk of major plus clinically relevant nonmajor bleeding, all bleeding, and gastrointestinal bleeding did significantly increase in the subgroup with CrCl <15 mL/min, and the risk of major plus clinically relevant nonmajor bleeding significantly increased in the subgroup with CrCl 15 to <30 mL/min. The J-RHYTHM registry also indicated that lower CrCl is not an independent predictor for major bleeding.^7^ In the ANAFIE registry, bleeding events were assessed using the International Society on Thrombosis and Haemostasis criteria, which may be the reason for the difference between this and previous findings.^5^^,^^7^ Possibly, hemoglobin levels were not adequately evaluated before and after the onset of hemorrhagic events. Alternatively, blood transfusions may have been given less frequently in elderly patients in actual clinical practice than in clinical trials.^26^ These points could explain this study’s low incidence of major bleeding. Additionally, all-cause mortality was very high in the CrCl <15 mL/min group and likely contributed to the lower incidence of major bleeding. There was a significant difference in the cumulative incidences of major bleeding among the 4 CrCl subgroups. However, in the multivariable analysis, renal dysfunction was not identified as an independent risk factor for major bleeding in this study. This result indicates that multiple comorbidities rather than reduced renal function may further contribute to major bleeding in elderly patients. Nevertheless, renal dysfunction was an independent risk factor for bleeding events, including major plus clinically relevant nonmajor bleeding, gastrointestinal bleeding, and all bleeding, particularly at CrCl levels <15 mL/min, and may be a predictor of bleeding events. Thus, for NVAF patients with renal dysfunction, anticoagulant therapy should be optimized to minimize the risk of bleeding.

### DOACs vs warfarin

Anticoagulant use was clearly different among the 4 renal function subgroups. As renal function decreased, the proportion of patients receiving anticoagulants overall became lower, and the administration of warfarin increased, but that of DOACs decreased (trend *P* < 0.001 for all). This decrease in the proportion of DOAC use, along with declining renal function, may be attributable to a preference among physicians to prescribe warfarin rather than DOACs for patients with renal dysfunction. Such decisions may be made considering the risk of bleeding and the limited data on the use of DOACs for elderly patients with renal dysfunction, especially patients with CrCl 15 to <30 mL/min. Another important finding was that the incidence rate of stroke/SEEs and ischemic stroke was lower with DOACs than with warfarin in patients with normal to moderately impaired renal function. These findings align with a previous analysis of the ENGAGE AF-TIMI 48 (Effective Anticoagulation With Factor Xa Next Generation in Atrial Fibrillation–Thrombolysis In Myocardial Infarction Study 48) trial,^22^ in which edoxaban exerted a consistent safety and efficacy benefit over warfarin in patients across all ranges of renal function. Furthermore, in the ROCKET-AF (The Rivaroxaban Once Daily Oral Direct Factor Xa Inhibition Compared with Vitamin K Antagonism for Prevention of Stroke and Embolism Trial in Atrial Fibrillation) study, patients with CrCl 30 to 49 mL/min had a numerically lower incidence of stroke/SEEs with rivaroxaban vs warfarin (HR: 0.84; 95% CI: 0.57-1.23).^18^ Our study also showed that in NVAF patients with moderate renal dysfunction (CrCl 30-<50 mL/min), the incidence rate of stroke/SEEs, stroke, ischemic stroke, major bleeding, ICH, cardiovascular death, all-cause death, net clinical outcomes, cardiac events, cardiovascular events, heart failure requiring hospitalization, and fractures was significantly lower in the DOAC group than in the warfarin group, which is consistent with previous reports.^8^^,^^22^^,^^27^

It is noteworthy that, although there was no difference in the risk of stroke/SEEs and major bleeding between DOACs and warfarin among NVAF patients with CrCl 15 to <30 mL/min, those receiving DOACs had a significantly lower risk of cardiac events, cardiovascular events, and heart failure requiring hospitalization than those receiving warfarin. Historically, phase 3 clinical trials have excluded patients with severe renal dysfunction (CrCl <30 mL/min). Although there is a tendency to select warfarin as the first choice for stroke/SEEs prevention for elderly patients with NVAF who have severe renal dysfunction (CrCl 15-<30 mL/min), the present results suggest that no OAC use significantly increased the risk of stroke/SEEs, all-cause death, and net clinical outcome compared with warfarin. On these bases, anticoagulant therapy with DOACs may be an appropriate treatment option for preventing events such as stroke/SEEs, all-cause death, and net clinical outcome in this population in real-world practice.

### Study limitations

The ANAFIE registry^26^ and this subanalysis included a Japanese-only population, limiting the generalizability of the results, and changes in warfarin and OAC prescriptions and doses were not accounted for. Additionally, nearly 19% of patients had unknown CrCl, which may have affected the incidence rates of outcome events. However, no clinically relevant differences were observed when the incidence rates of outcome events were compared between the unknown CrCl group and the 4 CrCl subgroups analyzed in this study (data not shown). The effects of changes in renal function over time were not investigated. CrCl calculations may not be the most accurate for assessing renal function in elderly patients. Modification of Diet in Renal Disease may be a better equation for elderly patients. Patients taking DOACs have a slower rate of renal function decline during treatment than those taking warfarin, which may have affected the incidence of events.^28^ Finally, there may have been other unknown confounders that might have affected the study results.

## Conclusions

In this subanalysis of the ANAFIE registry, the incidences of stroke/SEEs, major bleeding, major plus clinically relevant nonmajor bleeding, cardiovascular death, all-cause death, and net clinical outcomes increased as renal function declined in elderly NVAF patients (≥75 years of age). These findings imply that the relative effectiveness and safety of DOACs compared with warfarin in NVAF patients with normal renal function to moderate renal dysfunction (CrCl 30-<50 mL/min) are consistent with those previously reported.^8^^,^^22^^,^^27^ DOACs were effective and safe even in elderly NVAF patients with renal dysfunction (CrCl 15-<50 mL/min) compared with warfarin. Moreover, our data suggest that DOACs can be used safely in elderly NVAF patients with severe renal function decline (CrCl 15-<30 mL/min).

Perspectives **COMPETENCY IN MEDICAL KNOWLEDGE:** The findings of this subanalysis of the ANAFIE registry emphasize the importance of preventing stroke and cardiovascular outcomes among elderly patients with NVAF. Nearly half of this population had moderate-to-severe renal dysfunction (CrCl <50 mL/min), and the cumulative incidences of main clinical outcomes, including stroke/SEEs, major bleeding, cardiovascular death, all-cause death, and net clinical outcomes, increased as CrCl decreased. Lower CrCl is an independent risk factor for stroke/SEEs, major plus clinically relevant nonmajor bleeding, cardiovascular death, all-cause death, and net clinical outcomes in this population.

**TRANSLATIONAL OUTLOOK:** Our findings imply that DOACs may be used safely to prevent these events in elderly NVAF patients with severe renal function decline (CrCl >15 mL/min). Although generalizability was limited to Japanese and East Asian populations, we consider that these data translate into the routine care of very elderly patients with NVAF.

## Funding Support and Author Disclosures

This research was supported by Daiichi Sankyo. Dr Shimizu has received research funding from Daiichi Sankyo and Nippon Boehringer Ingelheim; and remuneration from Daiichi Sankyo, Pfizer Japan, Bristol Myers Squibb, Bayer, and Nippon Boehringer Ingelheim. Dr Yamashita has received research funding from Bristol Myers Squibb, Bayer, and Daiichi Sankyo; manuscript fees from Daiichi Sankyo and Bristol Myers Squibb; and remuneration from Daiichi Sankyo, Bayer, Pfizer Japan, and Bristol Myers Squibb. Dr Akao has received research funding from Bayer and Daiichi Sankyo; and remuneration from Bristol Myers Squibb, Nippon Boehringer Ingelheim, Bayer, and Daiichi Sankyo. Dr Atarashi has received remuneration from Daiichi Sankyo. Dr Ikeda has received research funding from Daiichi Sankyo and Bayer; and remuneration from Daiichi Sankyo, Bayer, Nippon Boehringer Ingelheim, and Bristol Myers Squibb. Dr Koretsune has received remuneration from Daiichi Sankyo, Bayer, and Nippon Boehringer Ingelheim. Dr Okumura has received remuneration from Nippon Boehringer Ingelheim, Daiichi Sankyo, Johnson & Johnson, and Medtronic. Dr Suzuki has received research funding from Daiichi Sankyo; and remuneration from Bristol Myers Squibb and Daiichi Sankyo. Dr Tsutsui has received research funding from Daiichi Sankyo and Nippon Boehringer Ingelheim; remuneration from Daiichi Sankyo, Bayer, Nippon Boehringer Ingelheim, and Pfizer Japan; scholarship funding from Daiichi Sankyo; and consultancy fees from Pfizer Japan, Bayer, and Nippon Boehringer Ingelheim. Dr Toyoda has received remuneration from Daiichi Sankyo, Otsuka, Novartis, Abbott, Bayer, and Bristol Myers Squibb. Dr Hirayama has participated in a course endowed by Boston Scientific Japan; received research funding from Daiichi Sankyo and Bayer; and received remuneration from Bayer, Daiichi Sankyo, Bristol Myers Squibb, and Nippon Boehringer Ingelheim. Dr Yasaka has received research funding from Nippon Boehringer Ingelheim; and remuneration from Nippon Boehringer Ingelheim, Daiichi Sankyo, Bayer, Bristol Myers Squibb, and Pfizer Japan. Dr Yamaguchi has served on the advisory board for Daiichi Sankyo; and received remuneration from Daiichi Sankyo and Bristol Myers Squibb. Dr Teramukai has received research funding from Nippon Boehringer Ingelheim; and remuneration from Daiichi Sankyo, Sanofi, Takeda, Chugai Pharmaceutical, Solasia Pharma, Bayer, Sysmex, Nipro, NapaJen Pharma, Gunze, Kaneka, Kringle Pharma, and Atworking. Mr Kimura, Dr Morishima, and Mr Takita are employees of Daiichi Sankyo. Dr Inoue has received remuneration from Daiichi Sankyo, Bristol Myers Squibb, and Nippon Boehringer Ingelheim.
