# Supplementary material for: Renal Function and Clinical Outcomes Among Elderly Patients With Nonvalvular Atrial Fibrillation From ANAFIE
Source: JACC Asia. 2023 Apr 11;3(3):475–87. doi: 10.1016/j.jacasi.2023.02.002 (PMC10308099; doi:10.1016/j.jacasi.2023.02.002)
Supplement: Supplemental Tables 1–5 [file mmc1.docx]

**SUPPLEMENTAL METHODS**

**Definitions of bleeding events per the International Society of Thrombosis and Haemostasis**

Major bleeding in non-surgical patients is defined as follows: fatal bleeding; symptomatic bleeding in a critical area or organ, such as intracranial, intraspinal, intraocular, retroperitoneal, intraarticular or pericardial, or intramuscular with compartment syndrome; and bleeding that causes a decrease in hemoglobin level of ≥2 g/dL (1.24 mmol/L) or leading to transfusion of ≥2 units of whole blood or red cells.

Major bleeding in surgical patients is defined as follows: fatal bleeding and/or bleeding that is symptomatic and occurs in a critical area or organ, such as intracranial, intraspinal, intraocular, retroperitoneal, pericardial, in a non-operated joint, or intramuscular with compartment syndrome, assessed in consultation with the surgeon; extrasurgical site bleeding causing a fall in hemoglobin level of ≥2 g/dL (1.24 mmol/L), or leading to transfusion of ≥2 units of whole blood or red cells, with temporal association within 24–48 h to the bleeding; surgical site bleeding that requires a second intervention (open, arthroscopic, endovascular) or a hemarthrosis of sufficient size to interfere with rehabilitation by delaying mobilization or delayed wound healing, resulting in prolonged hospitalization or deep wound infection; surgical site bleeding that is unexpected and prolonged and/or sufficiently large to cause hemodynamic instability, as assessed by the surgeon. There should be an associate fall in hemoglobin level of at least 2 g/dL (1.24 mmol/L), or transfusion, indicated by the bleeding, of at least two units of whole blood or red cells, with temporal association within 24 h to the bleeding. The data collection period is from the start of surgery until five half-lives after the last dose of the drug, with the longest half-life and the longest treatment period (in case of unequal active treatment durations).

Minor bleeding is defined as all non-major bleeds. Minor bleeds will be further divided into those that are clinically relevant and those that are not.

Clinically relevant minor bleed is defined as an acute or subacute clinically overt bleed that does not meet the criteria for a major bleed but prompts a clinical response, in that it leads to at least one of the following: a hospital admission for bleeding, physician-guided medical or surgical treatment for bleeding, or change in antithrombotic therapy (including interruption or discontinuation of study drug).

**Supplemental Table 1. Multivariable Analyses of Additional Outcomes by Renal Function**

| **Event by CrCl (mL/min)** | **N** | **Event (%)** | | **HR** | **(95% confidence interval)** | ***P*-value** |
| --- | --- | --- | --- | --- | --- | --- |
| **Stroke** |  |  |  |  |  |  |
| <15 | 404 | 17 | (4.2) | 1.30 | (0.78, 2.19) | 0.314 |
| 15 to <30 | 3465 | 131 | (3.8) | 1.28 | (1.00, 1.64) | 0.054 |
| 30 to <50 | 10 764 | 322 | (3.0) | 1.09 | (0.92, 1.30) | 0.316 |
| ≥50 | 11 569 | 294 | (2.5) |  | ref |  |
| **Ischemic stroke** |  |  |  |  |  |  |
| <15 | 404 | 15 | (3.7) | 1.53 | (0.88, 2.67) | 0.133 |
| 15 to <30 | 3465 | 106 | (3.1) | 1.36 | (1.02, 1.79) | 0.034 |
| 30 to <50 | 10 764 | 255 | (2.4) | 1.14 | (0.94, 1.39) | 0.179 |
| ≥50 | 11 569 | 223 | (1.9) |  | ref |  |
| **Hemorrhagic stroke** |  |  |  |  |  |  |
| <15 | 404 | 2 | (0.5) | 0.60 | (0.14, 2.58) | 0.496 |
| 15 to <30 | 3465 | 24 | (0.7) | 0.95 | (0.55, 1.65) | 0.859 |
| 30 to <50 | 10 764 | 67 | (0.6) | 0.91 | (0.63, 1.32) | 0.626 |
| ≥50 | 11 569 | 71 | (0.6) |  | ref |  |
| **SEE** |  |  |  |  |  |  |
| <15 | 404 | 4 | (1.0) | 14.66 | (3.14, 68.45) | <0.001 |
| 15 to <30 | 3465 | 7 | (0.2) | 2.23 | (0.61, 8.14) | 0.223 |
| 30 to <50 | 10 764 | 8 | (0.1) | 1.02 | (0.33, 3.18) | 0.976 |
| ≥50 | 11 569 | 6 | (0.1) |  | ref |  |
| **Minor bleeding** |  |  |  |  |  |  |
| <15 | 404 | 24 | (5.9) | 1.27 | (0.81, 1.99) | 0.303 |
| 15 to <30 | 3465 | 160 | (4.6) | 0.98 | (0.79, 1.21) | 0.858 |
| 30 to <50 | 10 764 | 469 | (4.4) | 1.00 | (0.87, 1.15) | 0.963 |
| ≥50 | 11 569 | 470 | (4.1) |  | ref |  |
| **Cardiac events** |  |  |  |  |  |  |
| <15 | 404 | 94 | (23.3) | 3.94 | (3.10, 5.01) | <0.001 |
| 15 to <30 | 3465 | 708 | (20.4) | 3.46 | (3.01, 3.98) | <0.001 |
| 30 to <50 | 10 764 | 999 | (9.3) | 1.88 | (1.68, 2.11) | <0.001 |
| ≥50 | 11 569 | 485 | (4.2) |  | ref |  |
| **Ischemic heart disease** |  |  |  |  |  |  |
| <15 | 404 | 9 | (2.2) | 4.33 | (2.06, 9.09) | <0.001 |
| 15 to <30 | 3465 | 42 | (1.2) | 2.50 | (1.61, 3.88) | <0.001 |
| 30 to <50 | 10 764 | 105 | (1.0) | 1.68 | (1.23, 2.29) | 0.001 |
| ≥50 | 11 569 | 77 | (0.7) |  | ref |  |
| **Myocardial infarction** |  |  |  |  |  |  |
| <15 | 404 | 4 | (1.0) | 5.60 | (1.78, 17.55) | 0.003 |
| 15 to <30 | 3465 | 28 | (0.8) | 4.89 | (2.53, 9.46) | <0.001 |
| 30 to <50 | 10 764 | 40 | (0.4) | 2.06 | (1.20, 3.54) | 0.009 |
| ≥50 | 11 569 | 23 | (0.2) |  | ref |  |
| **Heart failure requiring hospitalization** |  |  |  |  |  |  |
| <15 | 404 | 92 | (22.8) | 4.12 | (3.22, 5.27) | <0.001 |
| 15 to <30 | 3465 | 690 | (19.1) | 3.64 | (3.15, 4.22) | <0.001 |
| 30 to <50 | 10 764 | 921 | (8.6) | 1.93 | (1.71, 2.18) | <0.001 |
| ≥50 | 11 569 | 424 | (3.7) |  | ref |  |
| **Cardiac death and sudden death** |  |  |  |  |  |  |
| <15 | 404 | 9 | (2.2) | 13.70 | (5.31, 35.35) | <0.001 |
| 15 to <30 | 3465 | 40 | (1.2) | 6.79 | (3.33, 13.84) | <0.001 |
| 30 to <50 | 10 764 | 61 | (0.6) | 4.10 | (2.19, 7.66) | <0.001 |
| ≥50 | 11 569 | 13 | (0.1) |  | ref |  |
| **Cardiovascular events** |  |  |  |  |  |  |
| <15 | 404 | 103 | (25.5) | 2.96 | (2.37, 3.70) | <0.001 |
| 15 to <30 | 3465 | 784 | (22.6) | 2.72 | (2.41, 3.08) | <0.001 |
| 30 to <50 | 10 764 | 1248 | (11.6) | 1.61 | (1.46, 1.77) | <0.001 |
| ≥50 | 11 569 | 732 | (6.3) |  | ref |  |
| **Fractures and falls** |  |  |  |  |  |  |
| <15 | 404 | 64 | (15.8) | 1.10 | (0.84, 1.44) | 0.507 |
| 15 to <30 | 3465 | 591 | (17.1) | 1.20 | (1.06, 1.36) | 0.005 |
| 30 to <50 | 10 764 | 1233 | (11.5) | 1.03 | (0.94, 1.13) | 0.535 |
| ≥50 | 11 569 | 995 | (8.6) |  | ref |  |
| **Fractures** |  |  |  |  |  |  |
| <15 | 404 | 40 | (9.9) | 1.52 | (1.07, 2.15) | 0.020 |
| 15 to <30 | 3465 | 307 | (8.9) | 1.34 | (1.12, 1.60) | 0.002 |
| 30 to <50 | 10 764 | 609 | (5.7) | 1.13 | (0.99, 1.29) | 0.078 |
| ≥50 | 11 569 | 425 | (3.7) |  | ref |  |
| **Fall** |  |  |  |  |  |  |
| <15 | 404 | 58 | (14.4) | 1.12 | (0.84, 1.49) | 0.431 |
| 15 to <30 | 3465 | 535 | (15.4) | 1.22 | (1.07, 1.40) | 0.003 |
| 30 to <50 | 10 764 | 1106 | (10.3) | 1.04 | (0.94, 1.14) | 0.462 |
| ≥50 | 11 569 | 892 | (7.7) |  | ref |  |

*Confounding variables included in the model are as listed in Table 2.

Abbreviations: CrCl, creatinine clearance; HR, hazard ratio; SEE, systemic embolic events.

**Supplemental Table 2. Multivariable Analysis by Renal Function using the Fine–Gray Model with All-cause Death as a Competing Risk**

| **Event by CrCl (mL/min)** | **N** | **Event (%)** | | **HR** | **(95% confidence interval)** | ***P*-value** |
| --- | --- | --- | --- | --- | --- | --- |
| **Stroke/SEE** |  |  |  |  |  |  |
| <15 | 404 | 21 | (5.2) | 1.45 | (0.89, 2.36) | 0.133 |
| 15 to <30 | 3465 | 137 | (4.0) | 1.24 | (0.97, 1.60) | 0.085 |
| 30 to <50 | 10 764 | 329 | (3.1) | 1.08 | (0.91, 1.29) | 0.358 |
| ≥50 | 11 569 | 299 | (2.6) |  | ref |  |
| **Stroke** |  |  |  |  |  |  |
| <15 | 404 | 17 | (4.2) | 1.19 | (0.70, 2.01) | 0.525 |
| 15 to <30 | 3465 | 131 | (3.8) | 1.22 | (0.94, 1.57) | 0.132 |
| 30 to <50 | 10 764 | 322 | (3.0) | 1.08 | (0.91, 1.29) | 0.278 |
| ≥50 | 11 569 | 294 | (2.5) |  | ref |  |
| **Ischemic stroke** |  |  |  |  |  |  |
| <15 | 404 | 15 | (3.7) | 1.39 | (0.79, 2.44) | 0.256 |
| 15 to <30 | 3465 | 106 | (3.1) | 1.29 | (0.97, 1.71) | 0.079 |
| 30 to <50 | 10 764 | 255 | (2.4) | 1.13 | (0.93, 1.38) | 0.212 |
| ≥50 | 11 569 | 223 | (1.9) |  | ref |  |
| **Hemorrhagic stroke** |  |  |  |  |  |  |
| <15 | 404 | 2 | (0.5) | 0.56 | (0.12, 2.54) | 0.448 |
| 15 to <30 | 3465 | 24 | (0.7) | 0.90 | (0.50, 1.62) | 0.734 |
| 30 to <50 | 10 764 | 67 | (0.6) | 0.91 | (0.62, 1.32) | 0.607 |
| ≥50 | 11 569 | 71 | (0.6) |  | ref |  |
| **SEE** |  |  |  |  |  |  |
| <15 | 404 | 4 | (1.0) | 12.33 | (1.97, 77.05) | 0.007 |
| 15 to <30 | 3465 | 7 | (0.2) | 2.07 | (0.63, 6.83) | 0.233 |
| 30 to <50 | 10 764 | 8 | (0.1) | 1.00 | (0.31, 3.24) | 0.998 |
| ≥50 | 11 569 | 6 | (0.1) |  | ref |  |
| **Major bleeding** |  |  |  |  |  |  |
| <15 | 404 | 18 | (4.5) | 1.46 | (0.85, 2.51) | 0.173 |
| 15 to <30 | 3465 | 96 | (2.8) | 1.07 | (0.79, 1.45) | 0.677 |
| 30 to <50 | 10 764 | 216 | (2.0) | 0.94 | (0.76, 1.15) | 0.545 |
| ≥50 | 11 569 | 208 | (1.8) |  | ref |  |
| **Clinically relevant non-major bleeding** |  |  |  |  |  |  |
| <15 | 404 | 16 | (4.0) | 1.72 | (0.98, 3.03) | 0.058 |
| 15 to <30 | 3465 | 119 | (3.4) | 1.48 | (1.11, 1.98) | 0.008 |
| 30 to <50 | 10 764 | 212 | (2.0) | 0.99 | (0.80, 1.23) | 0.929 |
| ≥50 | 11 569 | 204 | (1.8) |  | ref |  |
| **Minor bleeding** |  |  |  |  |  |  |
| <15 | 404 | 24 | (5.9) | 1.13 | (0.71, 1.82) | 0.606 |
| 15 to <30 | 3465 | 160 | (4.6) | 0.93 | (0.75, 1.16) | 0.533 |
| 30 to <50 | 10 764 | 469 | (4.4) | 0.99 | (0.86, 1.13) | 0.856 |
| ≥50 | 11 569 | 470 | (4.1) |  | ref |  |
| **All bleeding** |  |  |  |  |  |  |
| <15 | 404 | 54 | (13.4) | 1.38 | (1.01, 1.89) | 0.045 |
| 15 to <30 | 3465 | 347 | (10.0) | 1.08 | (0.93, 1.26) | 0.320 |
| 30 to <50 | 10 764 | 848 | (7.9) | 0.97 | (0.87, 1.08) | 0.548 |
| ≥50 | 11 569 | 842 | (7.3) |  | ref |  |
| **Intracranial hemorrhage** |  |  |  |  |  |  |
| <15 | 404 | 8 | (2.0) | 0.89 | (0.41, 1.95) | 0.796 |
| 15 to <30 | 3465 | 57 | (1.6) | 0.90 | (0.62, 1.31) | 0.587 |
| 30 to <50 | 10 764 | 153 | (1.4) | 0.93 | (0.72, 1.18) | 0.540 |
| ≥50 | 11 569 | 152 | (1.3) |  | ref |  |
| **Gastrointestinal bleeding** |  |  |  |  |  |  |
| <15 | 404 | 26 | (6.4) | 1.49 | (0.95, 2.33) | 0.08 |
| 15 to <30 | 3465 | 160 | (4.6) | 1.09 | (0.87, 1.38) | 0.454 |
| 30 to <50 | 10 764 | 391 | (3.6) | 1.01 | (0.86, 1.18) | 0.933 |
| ≥50 | 11 569 | 375 | (3.2) |  | ref |  |
| **Major plus clinically relevant non-major bleeding** |  |  |  |  |  |  |
| <15 | 404 | 32 | (7.9) | 1.62 | (1.08, 2.43) | 0.029 |
| 15 to <30 | 3465 | 204 | (5.9) | 1.28 | (1.04, 1.59) | 0.022 |
| 30 to <50 | 10 764 | 417 | (3.9) | 0.98 | (0.84, 1.15) | 0.828 |
| ≥50 | 11 569 | 400 | (3.5) |  | ref |  |

*Confounding variables included in the model are as listed in Table 2.

Abbreviations: CrCl, creatinine clearance; HR, hazard ratio; SEE, systemic embolic events.

**Supplemental Table 3. Distribution of DOAC dose and PT-INR and TTR of warfarin by renal function**

| Item | | Overall | | No CrCl measured | | CrCl measured | | *P*-value* | By renal function | | | | | | | |  |
| --- | --- | --- | --- | --- | --- | --- | --- | --- | --- | --- | --- | --- | --- | --- | --- | --- | --- |
|  |  |  |  |  |  |  |  |  | <15 mL/min | | 15 to <30 mL/min | | 30 to <50 mL/min | | ≥50 mL/min | | *P*-value^#^ |
| DOAC | | 21 585 | | 3889 | | 17 696 | |  | 88 | | 1869 | | 7360 | | 8380 | |  |
|  | Standard dose | 3826 | (17.7) | 198 | (5.1) | 3628 | (20.5) |  | 0 | (0.0) | 9 | (0.5) | 503 | (6.8) | 3116 | (37.2) |  |
|  | Over dose | 698 | (3.2) | 31 | (0.8) | 667 | (3.8) |  | 0 | (0.0) | 36 | (1.9) | 425 | (5.8) | 206 | (2.5) |  |
|  | Reduced dose | 9548 | (44.2) | 506 | (13.0) | 9042 | (51.1) | <0.001 | 5 | (5.7) | 1550 | (82.9) | 5295 | (71.9) | 2192 | (26.2) | <0.001 |
|  | Under dose | 3630 | (16.8) | 103 | (2.6) | 3527 | (19.9) |  | 3 | (3.4) | 65 | (3.5) | 826 | (11.2) | 2633 | (31.4) |  |
|  | Off-label under dose | 795 | (3.7) | 105 | (2.7) | 690 | (3.9) |  | 0 | (0.0) | 154 | (8.2) | 309 | (4.2) | 227 | (2.7) |  |
| Warfarin | | 8233 | | 1613 | | 6620 | |  | 237 | | 1291 | | 2664 | | 2446 | |  |
|  | PT-INR | 2.0 ± 0.4 | | 2.0 ± 0.4 | | 2.0 ± 0.4 | | 0.888 | 1.9 ± 0.4 | | 1.9 ± 0.4 | | 2.0 ± 0.4 | | 2.0 ± 0.4 | | <0.001 |
|  | TTR | 75.5 ± 29.8 | | 77.0 ± 29.8 | | 75.1 ± 29.8 | | 0.051 | 63.9 ± 33.9 | | 69.8 ± 31.0 | | 75.9 ± 29.5 | | 78.1 ± 28.5 | | <0.001 |

Values are number (%) or mean ± standard deviation.

*With versus without CrCl measurement groups.

^#^ Among renal function subgroups.

Abbreviations: CrCl, creatinine clearance; DOAC, direct oral anticoagulant; PT-INR, prothrombin time-international normalized ratio; TTR, time in therapeutic range.

**Supplemental Table 4. Multivariable Analyses of Additional Outcomes by Renal Function and Type of Oral Anticoagulant**

| Event by type of anticoagulant | | CrCl 15 to <30 mL/min* | | | | | | CrCl 30 to <50 mL/min* | | | | | | CrCl ≥50 mL/min* | | | | | |
| --- | --- | --- | --- | --- | --- | --- | --- | --- | --- | --- | --- | --- | --- | --- | --- | --- | --- | --- | --- |
|  |  | N | Event (%) | |  | | | N | Event (%) | |  | | | N | Event (%) | |  | | |
|  |  |  |  |  | HR (95% confidence interval) | | *P*-value |  |  |  | HR (95% confidence interval) | | *P*-value |  |  |  | HR (95% confidence interval) | | *P*-value |
| **Stroke** | |  |  |  |  |  |  |  |  |  |  |  |  |  |  |  |  |  |  |
|  | Warfarin | 1291 | 48 | (3.7) |  | ref |  | 2664 | 98 | (3.7) |  | ref |  | 2446 | 80 | (3.3) |  | ref |  |
|  | No-OAC | 305 | 17 | (5.6) | 1.76 | (0.97, 3.18) | 0.062 | 737 | 28 | (3.8) | 1.22 | (0.78, 1.90) | 0.384 | 739 | 16 | (2.2) | 0.96 | (0.55, 1.68) | 0.885 |
|  | DOAC | 1869 | 66 | (3.5) | 0.91 | (0.62, 1.33) | 0.616 | 7360 | 196 | (2.7) | 0.76 | (0.59, 0.97) | 0.029 | 8380 | 198 | (2.4) | 0.77 | (0.59, 1.01) | 0.059 |
| **Ischemic stroke** | |  |  |  |  |  |  |  |  |  |  |  |  |  |  |  |  |  |  |
|  | Warfarin | 1291 | 41 | (3.2) |  | ref |  | 2664 | 77 | (2.9) |  | ref |  | 2446 | 62 | (2.5) |  | ref |  |
|  | No-OAC | 305 | 15 | (4.9) | 2.06 | (1.08, 3.91) | 0.028 | 737 | 26 | (3.5) | 1.45 | (0.90, 2.33) | 0.124 | 739 | 14 | (1.9) | 1.25 | (0.68, 2.30) | 0.477 |
|  | DOAC | 1869 | 50 | (2.7) | 0.82 | (0.54, 1.26) | 0.370 | 7,360 | 152 | (2.1) | 0.75 | (0.56, 0.99) | 0.040 | 8380 | 147 | (1.8) | 0.76 | (0.56, 1.03) | 0.082 |
| **Hemorrhagic stroke** | |  |  |  |  |  |  |  |  |  |  |  |  |  |  |  |  |  |  |
|  | Warfarin | 1291 | 7 | (0.5) |  | ref |  | 2664 | 20 | (0.8) |  | ref |  | 2446 | 18 | (0.7) |  | ref |  |
|  | No-OAC | 305 | 2 | (0.7) | 1.07 | (0.21, 5.51) | 0.938 | 737 | 3 | (0.4) | 0.65 | (0.19, 2.28) | 0.503 | 739 | 2 | (0.3) | 0.39 | (0.09, 1.74) | 0.216 |
|  | DOAC | 1869 | 15 | (0.8) | 1.29 | (0.50, 3.31) | 0.594 | 7360 | 44 | (0.6) | 0.84 | (0.49, 1.43) | 0.513 | 8380 | 51 | (0.6) | 0.82 | (0.47, 1.42) | 0.471 |
| **SEE** | |  |  |  |  |  |  |  |  |  |  |  |  |  |  |  |  |  |  |
|  | Warfarin | 1291 | 4 | (0.3) |  | ref |  | 2664 | 4 | (0.2) |  | ref |  | 2446 | 3 | (0.1) |  | ref |  |
|  | No-OAC | 305 | 1 | (0.3) | 2.38 | (0.19, 29.84) | 0.502 | 737 | 0 | (0.0) | 0.00 | (-、 -) | - | 739 | 0 | (0.0) | 0.00 | (-、 -) | - |
|  | DOAC | 1869 | 2 | (0.1) | 0.33 | (0.05, 2.48) | 0.284 | 7360 | 4 | (0.1) | 0.43 | (0.10, 1.88) | 0.264 | 8380 | 3 | (0.0) | 0.41 | (0.07, 2.26) | 0.303 |
| **Minor bleeding** | |  |  |  |  |  |  |  |  |  |  |  |  |  |  |  |  |  |  |
|  | Warfarin | 1291 | 58 | (4.5) |  | ref |  | 2664 | 127 | (4.8) |  | ref |  | 2446 | 111 | (4.5) |  | ref |  |
|  | No-OAC | 305 | 11 | (3.6) | 0.87 | (0.45, 1.70) | 0.689 | 737 | 21 | (2.8) | 0.57 | (0.35, 0.91) | 0.019 | 739 | 11 | (1.5) | 0.36 | (0.19, 0.68) | 0.002 |
|  | DOAC | 1869 | 91 | (4.9) | 1.08 | (0.77, 1.51) | 0.662 | 7360 | 321 | (4.4) | 0.93 | (0.76, 1.15) | 0.528 | 8380 | 348 | (4.2) | 0.96 | (0.77, 1.19) | 0.697 |
| **Cardiac events** | |  |  |  |  |  |  |  |  |  |  |  |  |  |  |  |  |  |  |
|  | Warfarin | 1291 | 307 | (23.8) |  | ref |  | 2664 | 301 | (11.3) |  | ref |  | 2446 | 114 | (4.7) |  | ref |  |
|  | No-OAC | 305 | 59 | (19.3) | 0.94 | (0.70, 1.25) | 0.657 | 737 | 52 | (7.1) | 0.73 | (0.54, 0.98) | 0.039 | 739 | 26 | (3.5) | 1.02 | (0.65, 1.58) | 0.937 |
|  | DOAC | 1869 | 342 | (18.3) | 0.80 | (0.68, 0.94) | 0.006 | 7,360 | 646 | (8.8) | 0.84 | (0.73, 0.97) | 0.016 | 8380 | 345 | (4.1) | 1.02 | (0.82, 1.26) | 0.881 |
| **Ischemic heart disease** | |  |  |  |  |  |  |  |  |  |  |  |  |  |  |  |  |  |  |
|  | Warfarin | 1291 | 20 | (1.5) |  | ref |  | 2664 | 22 | (0.8) |  | ref |  | 2446 | 20 | (0.8) |  | ref |  |
|  | No-OAC | 305 | 0 | (0.0) | 0.00 | (-、 -) | - | 737 | 9 | (1.2) | 1.37 | (0.61, 3.09) | 0.444 | 739 | 6 | (0.8) | 1.10 | (0.42, 2.84) | 0.852 |
|  | DOAC | 1,869 | 22 | (1.2) | 0.87 | (0.46, 1.63) | 0.657 | 7360 | 74 | (1.0) | 1.40 | (0.86, 2.27) | 0.175 | 8380 | 51 | (0.6) | 0.83 | (0.49, 1.42) | 0.502 |
| **Myocardial infarction** | |  |  |  |  |  |  |  |  |  |  |  |  |  |  |  |  |  |  |
|  | Warfarin | 1291 | 12 | (0.9) |  | ref |  | 2664 | 8 | (0.3) |  | ref |  | 2446 | 9 | (0.4) |  | ref |  |
|  | No-OAC | 305 | 0 | (0.0) | 0.00 | (-、 -) | - | 737 | 6 | (0.8) | 2.75 | (0.88, 8.59) | 0.082 | 739 | 1 | (0.1) | 0.47 | (0.05, 4.12) | 0.499 |
|  | DOAC | 1869 | 16 | (0.9) | 1.06 | (0.48, 2.31) | 0.893 | 7360 | 26 | (0.4) | 1.32 | (0.59, 2.95) | 0.505 | 8380 | 13 | (0.2) | 0.48 | (0.20, 1.15) | 0.100 |
| **Heart failure requiring hospitalization** | |  |  |  |  |  |  |  |  |  |  |  |  |  |  |  |  |  |  |
|  | Warfarin | 1291 | 297 | (23.0) |  | ref |  | 2,664 | 284 | (10.7) |  | ref |  | 2446 | 100 | (4.1) |  | ref |  |
|  | No-OAC | 305 | 59 | (19.3) | 0.97 | (0.73, 1.30) | 0.845 | 737 | 49 | (6.6) | 0.75 | (0.54, 1.02) | 0.066 | 739 | 21 | (2.9) | 0.99 | (0.61, 1.61) | 0.960 |
|  | DOAC | 1869 | 334 | (17.9) | 0.80 | (0.68, 0.94) | 0.007 | 7360 | 588 | (8.0) | 0.81 | (0.70, 0.93) | 0.004 | 8380 | 303 | (3.6) | 1.02 | (0.81, 1.29) | 0.843 |
| **Cardiac death and sudden death** | |  |  |  |  |  |  |  |  |  |  |  |  |  |  |  |  |  |  |
|  | Warfarin | 1291 | 12 | (0.9) |  | ref |  | 2664 | 16 | (0.6) |  | ref |  | 2446 | 2 | (0.1) |  | ref |  |
|  | No-OAC | 305 | 5 | (1.6) | 2.40 | (0.80, 7.24) | 0.119 | 737 | 4 | (0.5) | 1.09 | (0.35, 3.38) | 0.888 | 739 | 1 | (0.1) | 1.68 | (0.13, 21.74) | 0.690 |
|  | DOAC | 1869 | 23 | (1.2) | 1.31 | (0.63, 2.71) | 0.471 | 7360 | 41 | (0.6) | 0.98 | (0.54, 1.77) | 0.949 | 8380 | 10 | (0.1) | 1.95 | (0.41, 9.31) | 0.401 |
| **Cardiovascular events** | |  |  |  |  |  |  |  |  |  |  |  |  |  |  |  |  |  |  |
|  | Warfarin | 1291 | 338 | (26.2) |  | ref |  | 2664 | 369 | (13.9) |  | ref |  | 2446 | 175 | (7.2) |  | ref |  |
|  | No-OAC | 305 | 71 | (23.3) | 1.02 | (0.78, 1.34) | 0.859 | 737 | 74 | (10.0) | 0.85 | (0.66, 1.11) | 0.235 | 739 | 41 | (5.5) | 1.05 | (0.73, 1.49) | 0.803 |
|  | DOAC | 1869 | 375 | (20.1) | 0.78 | (0.67, 0.91) | 0.002 | 7360 | 805 | (10.9) | 0.85 | (0.75, 0.96) | 0.010 | 8380 | 516 | (6.2) | 0.97 | (0.81, 1.15) | 0.690 |
| **Fractures and falls** | |  |  |  |  |  |  |  |  |  |  |  |  |  |  |  |  |  |  |
|  | Warfarin | 1291 | 239 | (18.5) |  | ref |  | 2664 | 338 | (12.7) |  | ref |  | 2446 | 249 | (10.2) |  | ref |  |
|  | No-OAC | 305 | 51 | (16.7) | 0.98 | (0.71, 1.34) | 0.884 | 737 | 84 | (11.4) | 0.89 | (0.69, 1.13) | 0.336 | 739 | 47 | (6.4) | 0.60 | (0.44, 0.83) | 0.002 |
|  | DOAC | 1869 | 301 | (16.1) | 0.84 | (0.71, 1.00) | 0.051 | 7360 | 811 | (11.0) | 0.86 | (0.75, 0.97) | 0.019 | 8380 | 699 | (8.3) | 0.80 | (0.69, 0.93) | 0.003 |
| **Fractures** | |  |  |  |  |  |  |  |  |  |  |  |  |  |  |  |  |  |  |
|  | Warfarin | 1291 | 118 | (9.1) |  | ref |  | 2664 | 167 | (6.3) |  | ref |  | 2446 | 110 | (4.5) |  | ref |  |
|  | No-OAC | 305 | 32 | (10.5) | 1.24 | (0.82, 1.87) | 0.304 | 737 | 46 | (6.2) | 0.96 | (0.68, 1.35) | 0.800 | 739 | 20 | (2.7) | 0.54 | (0.33, 0.88) | 0.013 |
|  | DOAC | 1869 | 157 | (8.4) | 0.85 | (0.67, 1.09) | 0.208 | 7360 | 396 | (5.4) | 0.82 | (0.68, 0.98) | 0.030 | 8380 | 295 | (3.5) | 0.71 | (0.56, 0.89) | 0.003 |
| **Fall** | |  |  |  |  |  |  |  |  |  |  |  |  |  |  |  |  |  |  |
|  | Warfarin | 1291 | 219 | (17.0) |  | ref |  | 2664 | 297 | (11.1) |  | ref |  | 2446 | 224 | (9.2) |  | ref |  |
|  | No-OAC | 305 | 43 | (14.1) | 0.90 | (0.64, 1.26) | 0.531 | 737 | 76 | (10.3) | 0.92 | (0.71, 1.19) | 0.528 | 739 | 43 | (5.8) | 0.64 | (0.46, 0.89) | 0.009 |
|  | DOAC | 1869 | 273 | (14.6) | 0.84 | (0.70, 1.01) | 0.065 | 7360 | 733 | (10.0) | 0.89 | (0.78, 1.02) | 0.099 | 8380 | 625 | (7.5) | 0.80 | (0.69, 0.94) | 0.007 |

Warfarin group served as the reference.

*Confounding variables included in the model are as listed in Table 2.

Abbreviations: CI, confidence interval; CrCl, creatinine clearance; DOAC, direct oral anticoagulant; HR, hazard ratio; OAC, oral anticoagulant; SEE, systemic embolic events.

**Supplemental Table 5. Multivariable Analyses of Outcomes by Renal Function and by Type of Anticoagulant (excluding off-label doses of DOACs)**

| Event by type of anticoagulant | | CrCl 15 to <30 mL/min* | | | | | | CrCl 30 to <50 mL/min* | | | | | | CrCl ≥50 mL/min* | | | | | |
| --- | --- | --- | --- | --- | --- | --- | --- | --- | --- | --- | --- | --- | --- | --- | --- | --- | --- | --- | --- |
|  |  | N | Event (%) | |  | | | N | Event (%) | |  | | | N | Event (%) | |  | | |
|  |  |  |  |  | HR (95% confidence interval) | | P-value |  |  |  | HR (95% confidence interval) | | P-value |  |  |  | HR (95% confidence interval) | | P-value |
| **Stroke/SEE** | |  |  |  |  |  |  |  |  |  |  |  |  |  |  |  |  |  |  |
|  | Warfarin | 1291 | 51 | (4.0) |  | - |  | 2664 | 101 | (3.8) |  | - |  | 2446 | 83 | (3.4) |  | - |  |
|  | No-OAC | 305 | 18 | (5.9) | 1.83 | (1.03, 3.27) | 0.039 | 737 | 28 | (3.8) | 1.19 | (0.77, 1.86) | 0.433 | 739 | 16 | (2.2) | 0.92 | (0.53, 1.61) | 0.775 |
|  | DOAC | 1660 | 63 | (3.8) | 0.95 | (0.65, 1.40) | 0.807 | 7049 | 193 | (2.7) | 0.76 | (0.60, 0.97) | 0.030 | 8147 | 191 | (2.3) | 0.75 | (0.57, 0.97) | 0.029 |
| **Stroke** | |  |  |  |  |  |  |  |  |  |  |  |  |  |  |  |  |  |  |
|  | Warfarin | 1291 | 48 | (3.7) |  | - |  | 2664 | 98 | (3.7) |  | - |  | 2446 | 80 | (3.3) |  | - |  |
|  | No-OAC | 305 | 17 | (5.6) | 1.79 | (0.99, 3.24) | 0.055 | 737 | 28 | (3.8) | 1.22 | (0.78, 1.90) | 0.385 | 739 | 16 | (2.2) | 0.95 | (0.54, 1.66) | 0.854 |
|  | DOAC | 1660 | 62 | (3.7) | 0.99 | (0.67, 1.46) | 0.957 | 7049 | 189 | (2.7) | 0.77 | (0.60, 0.98) | 0.036 | 8147 | 190 | (2.3) | 0.77 | (0.59, 1.00) | 0.050 |
| **Ischemic stroke** | |  |  |  |  |  |  |  |  |  |  |  |  |  |  |  |  |  |  |
|  | Warfarin | 1291 | 41 | (3.2) |  | - |  | 2664 | 77 | (2.9) |  | - |  | 2446 | 62 | (2.5) |  | - |  |
|  | No-OAC | 305 | 15 | (4.9) | 2.11 | (1.11, 4.01) | 0.023 | 737 | 26 | (3.5) | 1.44 | (0.90, 2.32) | 0.131 | 739 | 14 | (1.9) | 1.24 | (0.67, 2.28) | 0.494 |
|  | DOAC | 1660 | 46 | (2.8) | 0.88 | (0.57, 1.36) | 0.561 | 7049 | 146 | (2.1) | 0.75 | (0.57, 0.99) | 0.046 | 8147 | 139 | (1.7) | 0.75 | (0.55, 1.01) | 0.062 |
| **Hemorrhagic stroke** | |  |  |  |  |  |  |  |  |  |  |  |  |  |  |  |  |  |  |
|  | Warfarin | 1291 | 7 | (0.5) |  | - |  | 2664 | 20 | (0.8) |  | - |  | 2446 | 18 | (0.7) |  | - |  |
|  | No-OAC | 305 | 2 | (0.7) | 1.08 | (0.21, 5.58) | 0.926 | 737 | 3 | (0.4) | 0.67 | (0.19, 2.34) | 0.526 | 739 | 2 | (0.3) | 0.39 | (0.09, 1.73) | 0.214 |
|  | DOAC | 1660 | 15 | (0.9) | 1.49 | (0.58, 3.82) | 0.413 | 7049 | 43 | (0.6) | 0.86 | (0.50, 1.47) | 0.580 | 8147 | 51 | (0.6) | 0.84 | (0.48, 1.46) | 0.533 |
| **SEE** | |  |  |  |  |  |  |  |  |  |  |  |  |  |  |  |  |  |  |
|  | Warfarin | 1291 | 4 | (0.3) |  | - |  | 2664 | 4 | (0.2) |  | - |  | 2446 | 3 | (0.1) |  | - |  |
|  | No-OAC | 305 | 1 | (0.3) | 6.21 | (0.30, 129.14) | 0.238 | 737 | 0 | (0.0) | 0.00 | (-、 -) | - | 739 | 0 | (0.0) | 0.00 | (-、 -) | - |
|  | DOAC | 1660 | 1 | (0.1) | 0.18 | (0.01, 2.93) | 0.228 | 7049 | 4 | (0.1) | 0.46 | (0.10, 2.01) | 0.301 | 8147 | 2 | (0.0) | 0.21 | (0.03, 1.64) | 0.136 |
| **Major bleeding** | |  |  |  |  |  |  |  |  |  |  |  |  |  |  |  |  |  |  |
|  | Warfarin | 1291 | 45 | (3.5) |  | - |  | 2664 | 74 | (2.8) |  | - |  | 2446 | 51 | (2.1) |  | - |  |
|  | No-OAC | 305 | 7 | (2.3) | 0.66 | (0.29, 1.52) | 0.333 | 737 | 15 | (2.0) | 0.74 | (0.41, 1.32) | 0.307 | 739 | 5 | (0.7) | 0.36 | (0.14, 0.93) | 0.035 |
|  | DOAC | 1660 | 40 | (2.4) | 0.69 | (0.44, 1.07) | 0.099 | 7049 | 124 | (1.8) | 0.65 | (0.49, 0.88) | 0.005 | 8147 | 149 | (1.8) | 0.92 | (0.67, 1.28) | 0.636 |
| **Major plus clinically relevant non-major bleeding** | |  |  |  |  |  |  |  |  |  |  |  |  |  |  |  |  |  |  |
|  | Warfarin | 1291 | 86 | (6.7) |  | - |  | 2664 | 112 | (4.2) |  | - |  | 2446 | 107 | (4.4) |  | ( |  |
|  | No-OAC | 305 | 15 | (4.9) | 0.65 | (0.37, 1.15) | 0.136 | 737 | 25 | (3.4) | 0.88 | (0.56, 1.38) | 0.578 | 739 | 9 | (1.2) | 0.28 | (0.14, 0.55) | <0.001 |
|  | DOAC | 1660 | 95 | (5.7) | 0.84 | (0.62, 1.14) | 0.275 | 7049 | 267 | (3.8) | 0.95 | (0.76, 1.19) | 0.669 | 8147 | 277 | (3.4) | 0.77 | (0.62, 0.97) | 0.029 |
| **Minor bleeding** | |  |  |  |  |  |  |  |  |  |  |  |  |  |  |  |  |  |  |
|  | Warfarin | 1291 | 58 | (4.5) |  | - |  | 2664 | 127 | (4.8) |  | - |  | 2446 | 111 | (4.5) |  | - |  |
|  | No-OAC | 305 | 11 | (3.6) | 0.91 | (0.47, 1.78) | 0.784 | 737 | 21 | (2.8) | 0.56 | (0.35, 0.91) | 0.018 | 739 | 11 | (1.5) | 0.36 | (0.19, 0.68) | 0.002 |
|  | DOAC | 1660 | 80 | (4.8) | 1.07 | (0.75, 1.51) | 0.715 | 7049 | 309 | (4.4) | 0.94 | (0.76, 1.16) | 0.582 | 8147 | 342 | (4.2) | 0.97 | (0.78, 1.20) | 0.761 |
| **All bleeding** | |  |  |  |  |  |  |  |  |  |  |  |  |  |  |  |  |  |  |
|  | Warfarin | 1291 | 135 | (10.5) |  | - |  | 2664 | 233 | (8.7) |  | - |  | 2446 | 212 | (8.7) |  | - |  |
|  | No-OAC | 305 | 24 | (7.9) | 0.71 | (0.45, 1.12) | 0.138 | 737 | 42 | (5.7) | 0.66 | (0.47, 0.92) | 0.015 | 739 | 19 | (2.6) | 0.31 | (0.19, 0.49) | <0. 001 |
|  | DOAC | 1660 | 169 | (10.2) | 0.98 | (0.78, 1.24) | 0.863 | 7049 | 550 | (7.8) | 0.93 | (0.79, 1.08) | 0.340 | 8147 | 599 | (7.4) | 0.87 | (0.74, 1.02) | 0.091 |
| **Intracranial hemorrhage** | |  |  |  |  |  |  |  |  |  |  |  |  |  |  |  |  |  |  |
|  | Warfarin | 1291 | 26 | (2.0) |  | - |  | 2664 | 56 | (2.1) |  | - |  | 2446 | 40 | (1.6) |  | - |  |
|  | No-OAC | 305 | 5 | (1.6) | 0.78 | (0.29, 2.11) | 0.623 | 737 | 7 | (0.9) | 0.50 | (0.22, 1.12) | 0.092 | 739 | 5 | (0.7) | 0.43 | (0.16, 1.11) | 0.080 |
|  | DOAC | 1660 | 25 | (1.5) | 0.69 | (0.39, 1.23) | 0.208 | 7049 | 87 | (1.2) | 0.61 | (0.43, 0.86) | 0.005 | 8147 | 106 | (1.3) | 0.81 | (0.56, 1.17) | 0.262 |
| **Gastrointestinal bleeding** | |  |  |  |  |  |  |  |  |  |  |  |  |  |  |  |  |  |  |
|  | Warfarin | 1291 | 61 | (4.7) |  | - |  | 2664 | 94 | (3.5) |  | - |  | 2446 | 91 | (3.7) |  | - |  |
|  | No-OAC | 305 | 8 | (2.6) | 0.53 | (0.25, 1.13) | 0.100 | 737 | 27 | (3.7) | 1.04 | (0.66, 1.62) | 0.873 | 739 | 13 | (1.8) | 0.50 | (0.28, 0.91) | 0.024 |
|  | DOAC | 1660 | 85 | (5.1) | 1.17 | (0.83, 1.64) | 0.376 | 7049 | 257 | (3.65) | 1.10 | (0.86, 1.40) | 0.439 | 8147 | 265 | (3.3) | 0.92 | (0.72, 1.17) | 0.495 |
| **Cardiac events** | |  |  |  |  |  |  |  |  |  |  |  |  |  |  |  |  |  |  |
|  | Warfarin | 1291 | 307 | (23.8) |  | - |  | 2664 | 301 | (11.3) |  | - |  | 2446 | 114 | (4.7) |  | - |  |
|  | No-OAC | 305 | 59 | (19.3) | 0.94 | (0.71, 1.26) | 0.695 | 737 | 52 | (7.1) | 0.72 | (0.53, 0.98) | 0.039 | 739 | 26 | (3.5) | 1.03 | (0.66, 1.60) | 0.905 |
|  | DOAC | 1660 | 309 | (18.6) | 0.82 | (0.69, 0.96) | 0.016 | 7049 | 614 | (8.7) | 0.83 | (0.72, 0.96) | 0.010 | 8147 | 329 | (4.0) | 1.00 | (0.80, 1.24) | 0.969 |
| **Ischemic heart disease** | |  |  |  |  |  |  |  |  |  |  |  |  |  |  |  |  |  |  |
|  | Warfarin | 1291 | 20 | (1.5) |  | - |  | 2664 | 22 | (0.8) |  | - |  | 2446 | 20 | (0.8) |  | - |  |
|  | No-OAC | 305 | 0 | (0.0) | 0.00 | (-、 -) | - | 737 | 9 | (1.2) | 1.35 | (0.60, 3.04) | 0.471 | 739 | 6 | (0.8) | 1.06 | (0.41, 2.75) | 0.906 |
|  | DOAC | 1660 | 21 | (1.3) | 0.94 | (0.49, 1.79) | 0.851 | 7049 | 70 | (1.0) | 1.37 | (0.84, 2.23) | 0.205 | 8147 | 49 | (0.6) | 0.82 | (0.48, 1.40) | 0.476 |
| **Myocardial infarction** | |  |  |  |  |  |  |  |  |  |  |  |  |  |  |  |  |  |  |
|  | Warfarin | 1291 | 12 | (0.9) |  | - |  | 2664 | 8 | (0.3) |  | - |  | 2446 | 9 | (0.4) |  | - |  |
|  | No-OAC | 305 | 0 | (0.0) | 0.00 | (-、 -) | - | 737 | 6 | (0.8) | 2.79 | (0.88, 8.80) | 0.081 | 739 | 1 | (0.1) | 0.49 | (0.06, 4.20) | 0.511 |
|  | DOAC | 1660 | 15 | (0.9) | 1.15 | (0.52, 2.54) | 0.737 | 7049 | 23 | (0.3) | 1.23 | (0.54, 2.80) | 0.617 | 8147 | 12 | (0.1) | 0.47 | (0.19, 1.14) | 0.096 |
| **Heart failure requiring hospitalization** | |  |  |  |  |  |  |  |  |  |  |  |  |  |  |  |  |  |  |
|  | Warfarin | 1291 | 297 | (23.0) |  | - |  | 2664 | 284 | (10.7) |  | - |  | 2446 | 100 | (4.1) |  | - |  |
|  | No-OAC | 305 | 59 | (19.3) | 0.98 | (0.73, 1.31) | 0.887 | 737 | 49 | (6.6) | 0.75 | (0.54, 1.02) | 0.067 | 739 | 21 | (2.8) | 1.01 | (0.62, 1.64) | 0.983 |
|  | DOAC | 1660 | 301 | (18.1) | 0.82 | (0.69, 0.97) | 0.017 | 7049 | 559 | (7.9) | 0.80 | (0.69, 0.92) | 0.003 | 8147 | 289 | (3.6) | 1.00 | (0.79, 1.27) | 0.980 |
| **Cardiac death and sudden death** | |  |  |  |  |  |  |  |  |  |  |  |  |  |  |  |  |  |  |
|  | Warfarin | 1291 | 12 | (0.9) |  | - |  | 2664 | 16 | (0.6) |  | - |  | 2446 | 2 | (0.1) |  | - |  |
|  | No-OAC | 305 | 5 | (1.6) | 2.48 | (0.82, 7.49) | 0.108 | 737 | 4 | (0.5) | 1.08 | (0.35, 3.38) | 0.889 | 739 | 1 | (0.1) | 1.40 | (0.11, 18.35) | 0.797 |
|  | DOAC | 1660 | 21 | (1.3) | 1.33 | (0.63, 2.80) | 0.451 | 7049 | 41 | (0.6) | 1.03 | (0.57, 1.86) | 0.928 | 8147 | 9 | (0.1) | 1.79 | (0.37, 8.73) | 0.471 |
| **Cardiovascular events** | |  |  |  |  |  |  |  |  |  |  |  |  |  |  |  |  |  |  |
|  | Warfarin | 1291 | 338 | (26.2) |  | - |  | 2664 | 369 | (13.9) |  | - |  | 2446 | 175 | (7.2) |  | - |  |
|  | No-OAC | 305 | 71 | (23.3) | 1.03 | (0.79, 1.35) | 0.812 | 737 | 74 | (10.0) | 0.85 | (0.66, 1.11) | 0.228 | 739 | 41 | (5.5) | 1.05 | (0.74, 1.50) | 0.792 |
|  | DOAC | 1660 | 340 | (20.5) | 0.81 | (0.69, 0.94) | 0.007 | 7049 | 767 | (10.9) | 0.84 | (0.74, 0.95) | 0.007 | 8147 | 492 | (6.0) | 0.95 | (0.79, 1.13) | 0.540 |
| **Cardiovascular death** | |  |  |  |  |  |  |  |  |  |  |  |  |  |  |  |  |  |  |
|  | Warfarin | 1291 | 74 | (5.7) |  | - |  | 2664 | 78 | (2.9) |  | - |  | 2446 | 27 | (1.1) |  | - |  |
|  | No-OAC | 305 | 23 | (7.5) | 1.60 | (0.98, 2.62) | 0.061 | 737 | 19 | (2.6) | 1.05 | (0.62, 1.78) | 0.858 | 739 | 5 | (0.7) | 0.95 | (0.35, 2.58) | 0.914 |
|  | DOAC | 1660 | 96 | (5.8) | 1.11 | (0.81, 1.52) | 0.518 | 7049 | 124 | (1.8) | 0.68 | (0.51, 0.90) | 0.008 | 8147 | 60 | (0.7) | 0.82 | (0.51, 1.30) | 0.395 |
| **All-cause deaths** | |  |  |  |  |  |  |  |  |  |  |  |  |  |  |  |  |  |  |
|  | Warfarin | 1291 | 204 | (15.8) |  | - |  | 2664 | 242 | (9.1) |  | - |  | 2446 | 95 | (3.9) |  | - |  |
|  | No-OAC | 305 | 72 | (23.6) | 1.74 | (1.31, 2.31) | <0.001 | 737 | 62 | (8.4) | 1.09 | (0.82, 1.47) | 0.548 | 739 | 31 | (4.2) | 1.37 | (0.90, 2.11) | 0.145 |
|  | DOAC | 1660 | 249 | (15.0) | 1.02 | (0.84, 1.23) | 0.866 | 7049 | 429 | (6.1) | 0.73 | (0.62, 0.86) | <0.001 | 8147 | 282 | (3.5) | 1.01 | (0.79, 1.28) | 0.949 |
| **Fractures and falls** | |  |  |  |  |  |  |  |  |  |  |  |  |  |  |  |  |  |  |
|  | Warfarin | 1291 | 239 | (18.5) |  | - |  | 2664 | 338 | (12.7) |  | - |  | 2446 | 249 | (10.2) |  | - |  |
|  | No-OAC | 305 | 51 | (16.7) | 0.97 | (0.71, 1.33) | 0.842 | 737 | 84 | (11.4) | 0.88 | (0.69, 1.13) | 0.335 | 739 | 47 | (6.4) | 0.60 | (0.44, 0.83) | 0.002 |
|  | DOAC | 1660 | 278 | (16.7) | 0.87 | (0.73, 1.04) | 0.132 | 7049 | 780 | (11.1) | 0.86 | (0.76, 0.98) | 0.025 | 8147 | 683 | (8.4) | 0.81 | (0.69, 0.94) | 0.005 |
| **Fractures** | |  |  |  |  |  |  |  |  |  |  |  |  |  |  |  |  |  |  |
|  | Warfarin | 1291 | 118 | (9.1) |  | - |  | 2664 | 167 | (6.3) |  | - |  | 2446 | 110 | (4.5) |  | - |  |
|  | No-OAC | 305 | 32 | (10.5) | 1.23 | (0.82, 1.86) | 0.316 | 737 | 46 | (6.2) | 0.96 | (0.68, 1.34) | 0.793 | 739 | 20 | (2.7) | 0.54 | (0.33, 0.88) | 0.014 |
|  | DOAC | 1660 | 144 | (8.7) | 0.87 | (0.68, 1.12) | 0.284 | 7049 | 385 | (5.5) | 0.83 | (0.69, 1.00) | 0.049 | 8147 | 292 | (3.6) | 0.72 | (0.58, 0.91) | 0.005 |
| **Fall** | |  |  |  |  |  |  |  |  |  |  |  |  |  |  |  |  |  |  |
|  | Warfarin | 1291 | 219 | (17.0) |  | - |  | 2664 | 297 | (11.1) |  | - |  | 2446 | 224 | (9.2) |  | - |  |
|  | No-OAC | 305 | 43 | (14.1) | 0.89 | (0.63, 1.25) | 0.489 | 737 | 76 | (10.3) | 0.92 | (0.70, 1.19) | 0.516 | 739 | 43 | (5.8) | 0.64 | (0.46, 0.90) | 0.009 |
|  | DOAC | 1660 | 252 | (15.2) | 0.87 | (0.72, 1.05) | 0.149 | 7049 | 704 | (10.0) | 0.90 | (0.78, 1.03) | 0.118 | 8147 | 609 | (7.5) | 0.81 | (0.69, 0.95) | 0.009 |
| **Net Clinical Outcome** | |  |  |  |  |  |  |  |  |  |  |  |  |  |  |  |  |  |  |
|  | Warfarin | 1291 | 259 | (20.1) |  | - |  | 2664 | 347 | (13.0) |  | - |  | 2446 | 181 | (7.4) |  | - |  |
|  | No-OAC | 305 | 85 | (27.9) | 1.61 | (1.24, 2.08) | <0.001 | 737 | 87 | (11.8) | 1.09 | (0.85, 1.40) | 0.492 | 739 | 48 | (6.5) | 1.12 | (0.80, 1.56) | 0.516 |
|  | DOAC | 1660 | 300 | (18.1) | 0.95 | (0.80, 1.12) | 0.530 | 7049 | 640 | (9.1) | 0.75 | (0.65, 0.85) | <0. 001 | 8147 | 523 | (6.4) | 0.95 | (0.80, 1.13) | 0.553 |

Data from the warfarin group was used as the reference.

*Confounding variables included in the model are as listed in Table 2.

Abbreviations: CI, confidence interval; CrCl, creatinine clearance; DOAC, direct oral anticoagulant; HR, hazard ratio; OAC, oral anticoagulant; SEE, systemic embolic events.
